# Species limits within the widespread Amazonian treefrog *Dendropsophus parviceps* with descriptions of two new species (Anura, Hylidae)

**DOI:** 10.3897/zookeys.726.13864

**Published:** 2018-01-08

**Authors:** C. Daniel Rivadeneira, Pablo J. Venegas, Santiago R. Ron

**Affiliations:** 1 Museo de Zoología, Escuela de Biología, Pontificia Universidad Católica del Ecuador, Av. 12 de Octubre y Roca, Aptdo. 17–01–2184, Quito, Ecuador; 2 Instituto de Ciencias Biológicas, Escuela Politécnica Nacional, Casilla 17-01-2759, Telefax: 2236690, Quito, Ecuador; 3 División de Herpetología-Centro de Ornitología y Biodiversidad (CORBIDI), Santa Rita N˚105 Of. 202, Urb. Huertos de San Antonio, Surco, Lima, Perú

**Keywords:** Advertisement call, Amazon Basin, cryptic species, integrative taxonomy, morphology, phylogeny

## Abstract

The genus *Dendropsophus* is one of the most speciose among Neotropical anurans and its number of described species is increasing. Herein, molecular, morphological, and bioacoustic evidence are combined to assess species limits within *D. parviceps*, a widely distributed species in the Amazon Basin. Phylogenetic relationships were assessed using 3040 bp sequences of mitochondrial DNA, genes 12S, ND1, and CO1. The phylogeny shows three well-supported clades. Bioacoustic and morphological divergence is congruent with those clades demonstrating that *Dendropsophus parviceps* is a species complex. *Dendropsophus parviceps* *sensu stricto* occurs in the Amazon basin of Ecuador, northern Peru, southern Colombia and northwestern Brazil. It is sister to two previously undescribed species, *D. kubricki* **sp. n.** from central Peru and *D. kamagarini* **sp. n.** from southern Peru, northeastern Bolivia, and northwestern Brazil. Genetic distances (uncorrected *p*, gene 12S) between *D. parviceps* and the new species is 3 to 4%. *Dendropsophus kamagarini* **sp. n.** can be distinguished from *D. parviceps* by having a prominent conical tubercle on the distal edge of the upper eyelid (tubercle absent in *D. parviceps*). *Dendropsophus kubricki* **sp. n.** differs from *D. parviceps* by having scattered low tubercles on the upper eyelids (smooth in *D. parviceps*). *Dendropsophus parviceps* and both new species differ from all their congeners by their small size (adult maximum SVL = 28.39 mm in females, 22.73 mm in males) and by having a bright orange blotch on the hidden areas of the shanks and under arms. The advertisement call of the two new species has lower dominant frequency relative to *D. parviceps*. Probable speciation modes are discussed. Available evidence indicates that ecological speciation along an elevation gradient is unlikely in this species complex.

## Introduction

The upper Amazon Basin harbors the highest diversity of amphibian species in the world (Bass et al. 2010; Duellman 1999). In the last decade, the use of genetic characters in amphibian taxonomy has helped to discover a large number of cryptic species through the upper and lower Amazon Basin (e.g., Almendáriz et al. 2014; Brown et al. 2008; Brown and Twomey 2009; Caminer and Ron 2014; Elmer and Cannatella 2008; Fouquet et al. 2015; Moravec et al. 2014; Páez-Vacas et al. 2010; Rivera-Correa and Orrico 2013; Rojas et al. 2015; Rojas et al. 2016; Ron et al. 2012; Twomey and Brown 2008). The use of genetic characters in combination with morphological and bioacoustic evidence allows unambiguous delimitation of species under the evolutionary species concept (de Queiroz 1998; de Queiroz 2007; Padial et al. 2009).

*Dendropsophus* Fitzinger 1843 is the most speciose genus of hylid frogs in the Neotropics. Currently it has 102 formally described species (Frost 2017). The few systematics studies of *Dendropsophus* that have included genetic evidence have resulted in the discovery of a large number of undescribed species (e.g., Fouquet et al. 2015; Gehara et al. 2014; Motta et al. 2012; Rivera-Correa and Orrico 2013). These studies underscore the need of genetics-based taxonomic reviews in the genus *Dendropsophus*.

Within *Dendropsophus*, some species groups are not monophyletic and relationships among their species are unclear (Faivovich et al. 2005; Fouquet et al. 2011; Fouquet et al. 2015; Motta et al. 2012; Pyron and Wiens 2011; Wiens et al. 2010). One of them is the *Dendropsophus parviceps* species group (*sensu* Faivovich et al. 2005). It contains 15 species (Frost 2017): *D. bokermanni* (Goin, 1960), *D. brevifrons* (Duellman and Crump 1974), *D. counani* Fouquet et al., 2015, *D. frosti* Motta et al., 2012, *D. giesleri* (Mertens, 1950), *D. grandisonae* (Goin, 1966), *D. koechlini* (Duellman & Trueb, 1989), *D. luteoocellatus* (Roux, 1927), *D. microps* (Peters, 1872), *D. parviceps* (Boulenger, 1882), *D. pauiniensis* (Heyer, 1977), *D. ruschii* (Weygoldt & Peixoto, 1987), *D. schubarti* (Bokermann, 1963), *D. subocularis* (Dunn, 1934), and *D. timbeba* (Martins & Cardoso, 1987). Eleven species of the group occur in the Amazon basin: *D. bokermanni*, *D. brevifrons*, *D. counani*, *D. frosti*, *D. grandisonae*, *D. koechlini*, *D. luteoocellatus*, *D. parviceps*, *D. pauiniensis*, *D. schubarti*, and *D. timbeba*. Of the remaining, three occur in the Brazilian Atlantic Forest (*D. giesleri*, *D. microps*, and *D. ruschii*) and one in the lowlands of eastern Darien (Panama) and northwestern Colombia (*D. subocularis*). A recent phylogeny by Fouquet et al. (2015) recovered the “*D. parviceps* clade” with strong support. The clade included *D. bokermanni*, *D. brevifrons*, *D. counani*, *D. frosti*, *D. koechlini*, and *D. parviceps*, where *D. koechlini* is sister to a clade of the remaining species.

*Dendropsophus parviceps* is a small treefrog described by Boulenger (1882) from “Sarayacu” (= Sarayaku), Pastaza Province, Ecuador. *Dendropsophus parviceps* is characterized by having a short and truncate snout, one bar below the orbit, dark brown markings on dorsum, and a bright orange blotch on the proximal ventral surface of the shanks (Duellman and Crump 1974; Duellman 1978). *Dendropsophus parviceps* is widely distributed in the Amazon Basin of Brazil, Venezuela, Colombia, Ecuador, Peru, and Bolivia (Frost 2017). Its elevation range is 186–1600 m (Ron and Read 2012). Duellman (2005) suggested that *D. parviceps* from southern Peru might not be conspecific with *D. parviceps* *sensu stricto* because Ecuadorian populations are smaller and lack a prominent tubercle on the edge of the upper eyelid. Until now a comprehensive taxonomic review of *D. parviceps* has been missing. Herein we assess the species limits within “*D. parviceps*” with genetic, morphological, and bioacoustic data. Our results reveal the existence of two new species that we describe here.

## Materials and methods

### Morphological analyses

Frogs were fixed in 10% formalin and preserved in 70% ethanol. Examined specimens, listed in Appendix 1, are housed at Museo de Zoología, Pontificia Universidad Católica del Ecuador (QCAZ), and the División de Herpetología, Centro de Ornitología y Biodiversidad (CORBIDI), Lima, Peru. We also examined the holotype of *Dendropsophus parviceps* at the Natural History Museum (NHM), London, UK.

The following measurements were made with digital calipers (nearest 0.01 mm) for adult specimens, following Cisneros-Heredia and McDiarmid (2007), Duellman (1970), and Motta et al. (2012):

**SVL** snout-vent length; **HW** head width; **HL** head length; **END** eye to nostril distance; **IN** internarial distance; **FL** femur length; **TL** tibia length; **FL** foot length.

A total of 159 specimens from Ecuador and Peru was measured. Webbing formulae are described following Cisneros-Heredia and McDiarmid (2007). Sex was determined by size differences (mean male SVL = 16.4 mm and mean female SVL = 22.5 mm) and by gonadal inspection. Description of coloration in life is based on field notes and digital color photographs.

Principal Components Analysis (PCA) was used to assess morphometric differentiation between species. Prior to analysis, all morphometric variables were log-transformed to achieve a normal distribution. To remove the effect of body size, the PCA was applied to the residuals of the linear regressions between the SVL and the morphometric variables, for males and females separately. Only principal components with eigenvalues > 1 were retained. We compared morphometric variables between species with Student’s t-test. All analyses were performed using JMP® 9.0.1 (SAS Institute 2010).

### Bioacoustic analyses

Recordings were made with two digital recorders Olympus LS-10 and Marantz professional PMD620MKII Handheld Solid State Recorder attached to a directional microphone Sennheiser K6–ME67. We also included published recordings from Peru, Tambopata (Cocroft et al. 2001), and Bolivia, Cobija (Márquez et al. 2002). Recordings are deposited at the on QCAZ collection.

Calls were analyzed using software Raven 1.3 (Charif et al. 2004) at a sampling rate of 44100 Hz and a resolution of 16 bits. Spectral parameters were obtained using a Fast Fourier Transformation (FFT) of 4096 points, a frequency resolution of 10.8 Hz, window type Hann and filter bandwidth of 52.2 Hz.

Terminology for call parameters follows Köhler et al. (2017) and Toledo et al. (2015). We measured the following variables: (1) call duration: time from the beginning to the end of the call; (2) note duration: time from beginning to end of the note; (3) rise time: time from the beginning of the note to the point of maximum amplitude; (4) number of pulses: number of pulses in the note; (5) pulse rate: number of pulses per note duration; (6) interval between notes: time from the end of one note to the beginning of the next; (7) dominant frequency: frequency with the most energy, measured along the entire call; (8) initial frequency: frequency at the beginning of the note; and (9) final frequency: frequency at the end of the note. If available, several calls or notes were analyzed per individual to calculate an individual average.

The calls of members of the *Dendropsophus parviceps* group (*sensu* Fouquet et al. 2015) consist of one high-pitched pulsed trill followed or not by a series of clicks: *D. counani* (Fouquet et al. 2015), *D. bokermanni* (Duellman and Crump 1974; Fouquet et al. 2015; Read and Ron 2012), *D. brevifrons* (Fouquet et al. 2015; Read and Ron 2011), and *D. koechlini* (Duellman and Trueb 1989; Fouquet et al. 2015). Therefore, we used a note-centered approach to define what is considered a call and a note (*sensu* Köhler et al. 2017).

A Principal Components Analysis (PCA) was conducted to evaluate call differentiation between species. We also performed Student’s t-test to assess differences between species in the acoustic variables. For the PCA, only components with eigenvalues > 1 were retained. All statistical analyses were performed using JMP® 9.0.1 (SAS Institute 2010). Some recordings did not have temperature registered but temperature variation in equatorial rainforests at night is low (Duellman 1978) and therefore unlikely to severely influence the analyses.

### Phylogenetic analyses

***DNA extraction, amplification, and sequencing***

Total DNA was extracted from muscle and liver preserved in 95% ethanol or tissue storage buffer using guanidine–thiocyanate extraction protocol of M. Fujita (unpublished). Polymerase chain reaction (PCR) was used to amplify the mitochondrial genes 12S rRNA (12S), Cytochrome Oxidase 1 (CO1), and a continuous fragment of 16S (partial sequence), tRNA^Leu^, NADH dehydrogenase subunit 1 (ND1), tRNA^Ile^, and tRNA^Gln^. PCRs were performed in 25 μl reactions using 2.5 μl of PCR buffer, 1.5 μl MgCl_2_, 0.5 μl of each primer, 0.5 μl of each dNTP, 0.25 μl of Taq polymerase, 1 U of DNA, and 18.25 μl dH_2_O. Primers are listed in Table 1. PCR amplification was carried under standard protocols. PCR products were visualized in 1% agarose gel, and primers residues and dNTPs were removed from PCR products using ExoSAP-It purification. Amplified products were sequenced by the Macrogen Sequencing Team (Macrogen Inc., Seoul, Korea).

**Table 1.** Primers used in this study.

Gene	Primer	Primer sequence (5’–3’)	Source
12S	tPhe-frog	ATAGCRCTGAARAYGCTRAGATG	Wiens et al. (2005)
	tVal-frog	TGTAAGCGARAGGCTTTKGTTAAGCT	Wiens et al. (2005)
ND1	16S-frog	TTACCCTRGGGATAACAGCGCAA	Wiens et al. (2005)
	WL384	GAGATWGTTTGWGCAACTGCTCG	Moen and Wiens (2009)
	WL379b	GCACTAGCAATAATTATYTGAACBCC	This study
	tMet-frog	TTGGGGTATGGGCCCAAAAGCT	Wiens et al. (2005)
CO1	COI-BirdF1	TTCTCCAACCACAAAGACATTGGCAC	Hebert et al. (2004)
	COI-BirdR2	ACGTGGGAGATAATTCCAAATCCTGG	Hebert et al. (2004)
	LCO1490	GGTCAACAAATCATAAAGATATTGG	Folmer et al. (1994)
	dgHCO2198	TAAACTTCAGGGTGACCAAARAAYCA	Folmer et al. (1994)

New sequences were obtained from 61 specimens from the upper Amazon Basin of Ecuador and Peru. A sequence of *Dendropsophus parviceps* available in GenBank published by Faivovich et al. (2005) from Brazil (Acre) was also downloaded. Sequences of three closely related species, *D. brevifrons*, *D. frosti*, and *D. koechlini* were also included. *Dendropsophus marmoratus* and *Xenohyla truncata* were used as outgroups. Sequences of *D. brevifrons*, *D. frosti*, and *X. truncata* were published by Fouquet et al. (2015), Motta et al. (2012), and Faivovich et al. (2005), respectively.

Sequences were assembled and aligned in Geneiuos Pro v5.4.6 (Kearse et al. 2012) using the MAFFT plugin under the L-INS-i algorithm (Katoh et al. 2002). Manual adjustments to the alignment were made using Mesquite v3.04 (Maddison and Maddison 2015). ND1 and CO1 gene sequences were translated into amino acids in Mesquite to confirm the alignment and verify the absence of stop codons.

***Phylogeny***

Phylogenetic relationships were inferred using Maximum likelihood (ML) with software GARLI v2.0 (Zwickl 2006) and Bayesian inference with MrBayes v3.1.2 (Ronquist et al. 2012). The best partition strategy and the best-fit substitution model of DNA evolution for each partition were selected using PartitionFinder v1.1.0 (Lanfear et al. 2012) according to the Bayesian Information Criterion (BIC). We defined nine *a priori* partitions: 12S, 16S, tRNAs, and one partition for each codon position of ND1 and CO1.

Maximum likelihood analyses were performed with ten replicates starting from stepwise addition trees (streefname = stepwise). Other GARLI settings were set to default values (Zwickl 2006). Bootstrap support was evaluated through 500 replicates. The 50% majority rule consensus for the bootstrap trees was obtained with Mesquite v3.04 (Maddison and Maddison 2015). Bayesian analyses were performed with two searches of 35 × 10^7^ generations each with four Markov chains and trees sampled every 5000 generations; stationarity and convergence were assessed in Tracer v1.6 (Rambaut et al. 2014) examining the standard deviation of split frequencies and plotting the –lnL per generation. Trees generated before stationarity were discarded as burn-in. Additionally, pairwise genetic distances (uncorrected *p*) were calculated for 12S using MEGA 6.0 (Tamura et al. 2013).

## Results

### Phylogenetic relationships

The total alignment of concatenated DNA sequences had 3040 base pairs from mitochondrial markers 12S rRNA (~895 bp), small fragment of 16S rRNA (~282 bp), portions of tRNA (~215 bp), ND1 (~961 bp) and CO1 (~687 bp) from 70 individuals. Genes sequenced and GenBank accession numbers are listed in Appendix 2. The best partition strategy and the best-fit model for each partition are shown in Table 2.

**Table 2.** Partition strategy and the best-fit model of substitution for each partition block used in phylogenetic analyses.

**Partition**	**Best Model**	**Partition blocks**
1	GTR + G	12S, tRNA, ND1, 1^st^ position
2	K80 + I + G	16S
3	HKY + I	ND1, 2^nd^ position
4	GTR + I	ND1, 3^rd^ position, CO1, 3^rd^ position
5	K80 + I	CO1, 1^st^ position
6	F81	CO1, 2^nd^ position

**Figure 1.**
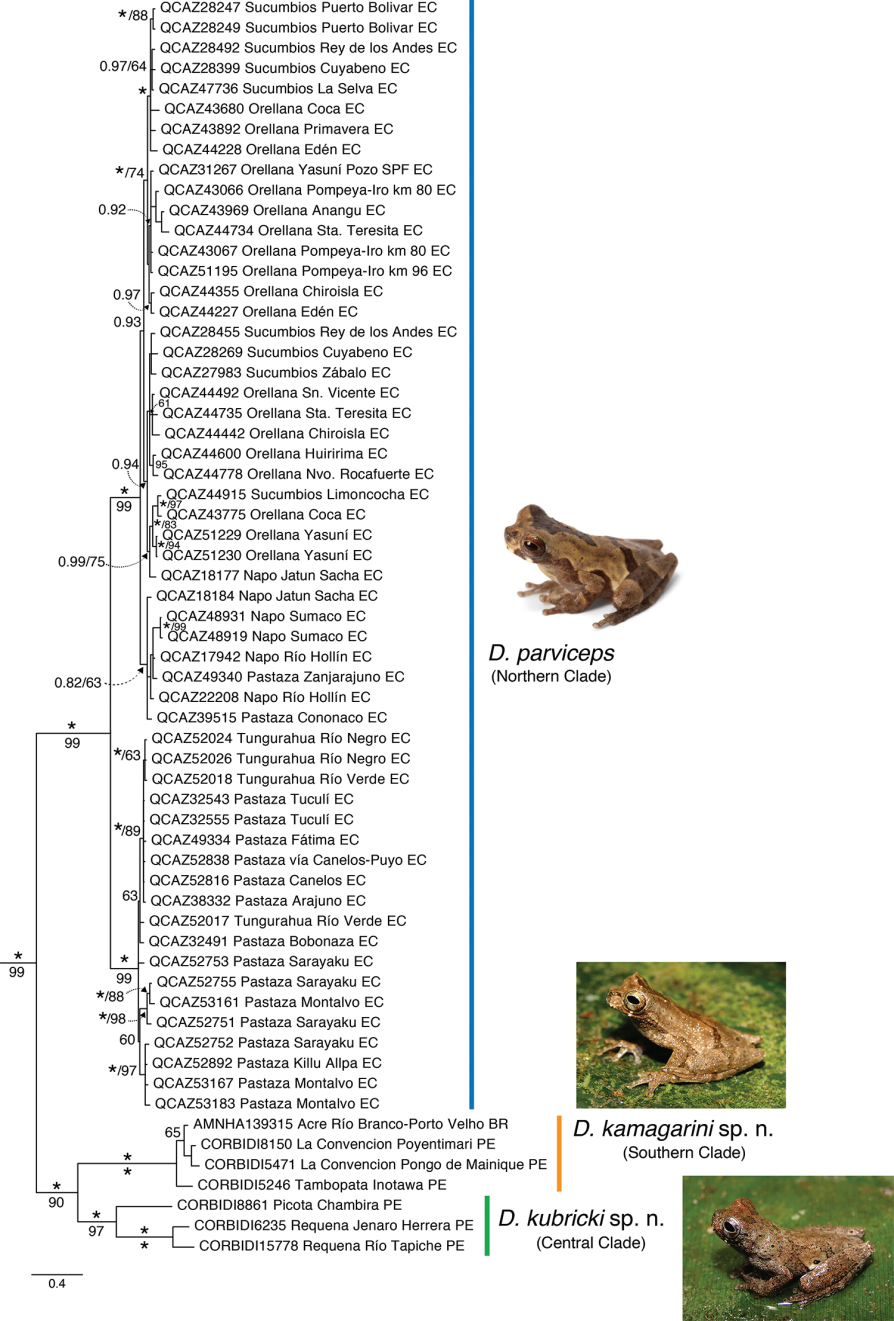
Bayesian consensus phylogeny of *Dendropsophus parviceps* species complex based on 3040 bp of mtDNA. Node support is indicated with Bayesian posterior probabilities (pp) above branches and non-parametric bootstrap support below. Asteriks denote nodes with pp = 1 and bootstrap values = 100%. Outgroups, bootstrap values < 60%, and pp < 0.8 are not shown. Museum number and locality are provided for each sample. Abbreviations: BR = Brazil, PE = Peru, and EC = Ecuador.

The phylogenetic relationships strongly support *Dendropsophus parviceps* as monophyletic (posterior probability, pp = 1 and bootstrap = 99) (Fig. 1). There are three clades within *D. parviceps*, each strongly supported. One clade is distributed in southern Peru (e.g., Madre de Dios and Cusco regions) and northwest Brazil (Acre); we refer to this clade as the “Southern Clade” hereafter. The second clade is distributed in northern and central Peru (e.g., Sierra del Divisor, Río Tapiche, and Chambira) (“Central Clade” hereafter). The third clade is distributed in eastern Ecuador (called “Northern Clade” hereafter). Maximum pairwise uncorrected genetic distance for 12S between the Central Clade and the Southern Clade is 2.8%, between the Northern Clade and the Central Clade is 3.2% and between the Northern Clade and the Southern Clade is 3.7%.

Mean *p* genetic distance within the Central Clade is 1.3% (range 0–1.3%) while within the Southern Clade is 0.07% (range 0–0.15%). The Northern Clade is divided in two subclades also with high support (pp = 1 and bootstrap = 99%). One subclade includes populations in the northern Amazon of Ecuador on the Napo River while the other includes populations in the central and southern Amazon of Ecuador (Fig. 1). Mean genetic divergence between these two subclades is 0.8% (range 0.4–1.2%) suggesting that they are deep conspecific lineages.

### Morphological comparisons

**Figure 2.**
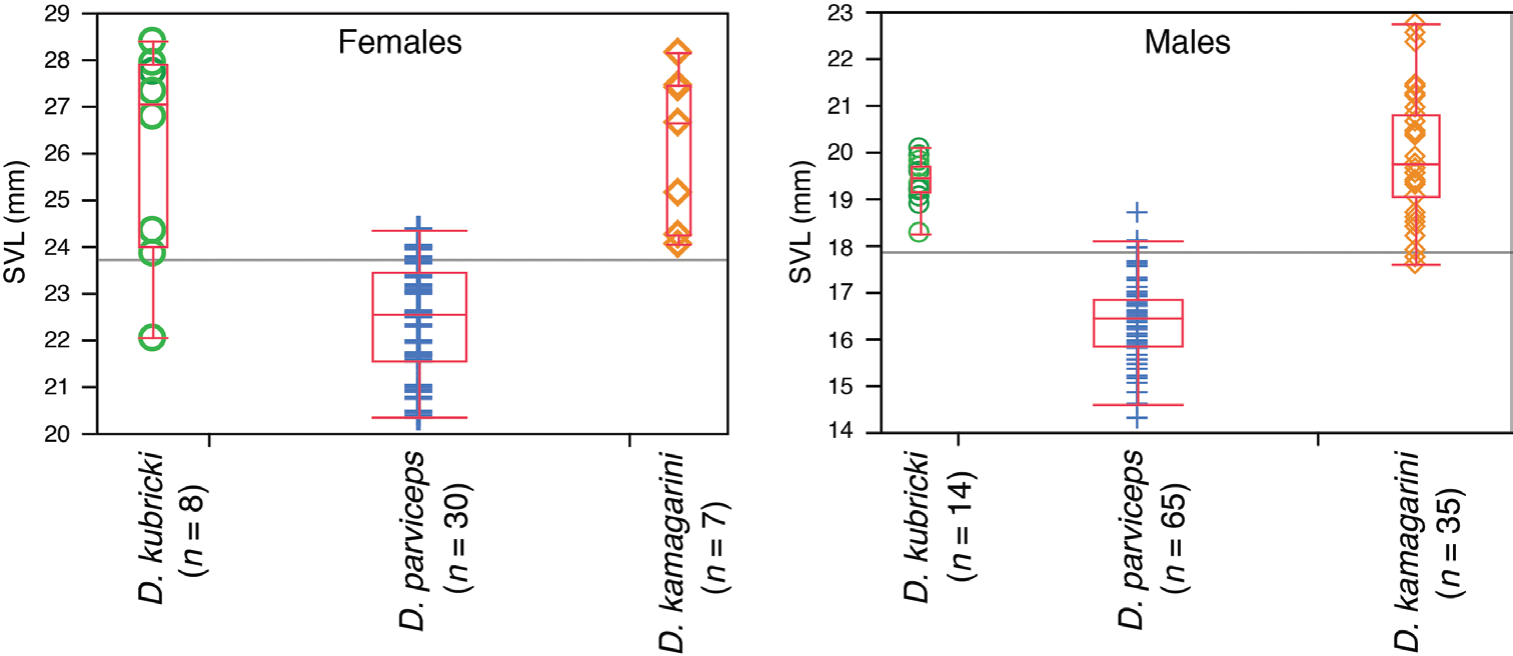
Boxplots for snout-vent length of adults of *Dendropsophus parviceps* (Northern Clade), *D. kamagarini* sp. n. (Southern Clade), and *D. kubricki* sp. n. (Central Clade). The line in the middle of the box represents the median, and the lower and upper ends of the box are the 25% and 75% quartiles, respectively; whiskers represent the minimum and maximum values. Each specimen is shown with a symbol.

Morphometric variables from adults are summarized in Table 3. The Northern Clade has smaller size than the Southern and Central clades (Fig. 2; Table 3; Student’s t test Northern Clade vs. Southern Clade, *t* = 16.18, df = 98, *p* < 0.001 for males and *t* = 6.85, df = 35, *p* < 0.001 for females; Student’s t test Northern Clade vs. Central Clade, *t* = –12.86, df = 77, *p* < 0.001 for males and *t* = –6.08, df = 36, *p* < 0.001 for females).

**Table 3.** Descriptive statistics for morphometric measurements of adult *Dendropsophus parviceps* (Northern Clade), *D. kamagarini* sp. n. (Southern Clade), and *D. kubricki* sp. n. (Central Clade). Mean ± SD is given with range below. Abbreviations are: SVL = snout-vent length; HW = head width; HL = head length; END = eye to nostril distance; IN = internarial distance between the nostrils; FL = femur length; TL = tibia length; FL = foot length. All measurements are in mm.

	*Dendropsophus parviceps*		*Dendropsophus kamagarini* sp. n.		*Dendropsophus kubricki* sp. n.	
	Males *n* = 65	Females *n* = 30	Males *n* = 35	Females *n* = 7	Males *n* = 14	Females *n* = 8
**SVL**	16.4 ± 0.84 (14.3−18.7)	22.5 ± 1.17 (20.3−24.4)	19.9 ± 1.33 (17.6−22.7)	26.1 ± 1.67 (24.0−28.1)	19.4 ± 0.48 (18.3−20.1)	26.0 ± 2.33 (22.0−28.4)
**HW**	5.2 ± 0.30 (4.6−5.9)	6.8 ± 0.32 (6.2−7.4)	6.3 ± 0.40 (5.5−7.0)	8.2 ± 0.50 (7.3−8.8)	6.4 ± 0.24 (6.0−6.7)	8.2 ± 0.85 (6.8−9.3)
**HL**	4.9 ± 0.36 (4.2−5.8)	6.1 ± 0.54 (5.3−7.5)	6.2 ± 0.34 (5.4−6.8)	7.7 ± 0.41 (6.9−8.1)	6.3 ± 0.29 (5.9−7.0)	7.5 ± 0.40 (7.0−8.2)
**END**	1.7 ± 0.14 (1.4−2.2)	2.1 ± 0.17 (1.9−2.4)	2.0 ± 0.16 (1.7−2.3)	2.4 ± 0.17 (2.4−2.6)	2.1 ± 0.26 (1.8−2.7)	2.7 ± 0.33 (2.3−3.3)
**IN**	1.6 ± 0.14 (1.3−2.0)	2.0 ± 0.18 (1.7−2.4)	1.8 ± 0.16 (1.5−2.2)	2.2 ± 0.12 (2.0−2.4)	1.8 ± 0.11 (1.5−2.0)	2.3 ± 0.22 (2.0−2.7)
**FL**	7.8 ± 0.48 (6.6−8.9)	11.2 ± 0.67 (9.9−12.6)	9.8 ± 0.67 (8.5−11.3)	13.1 ± 0.74 (12.1−14.0)	9.7 ± 0.52 (8.9−10.7)	12.7 ± 0.69 (11.9−13.6)
**TL**	8.6 ± 0.49 (7.2−9.8)	12.2 ± 0.65 (10.7−13.5)	10.6 ± 0.74 (9.0−11.8)	14.1 ± 0.56 (13.3−15.0)	10.4 ± 0.41 (9.8−11.1)	13.8 ± 1.08 (12.3−15.5)
**FL**	6.5 ± 0.49 (5.4−7.7)	9.1 ± 0.88 (7.3−10.6)	8.3 ± 0.65 (7.0−9.4)	11.3 ± 0.81 (10.3−12.6)	7.9 ± 0.38 (7.3−8.8)	10.4 ± 0.55 (9.6−11.5)

**Figure 3.**
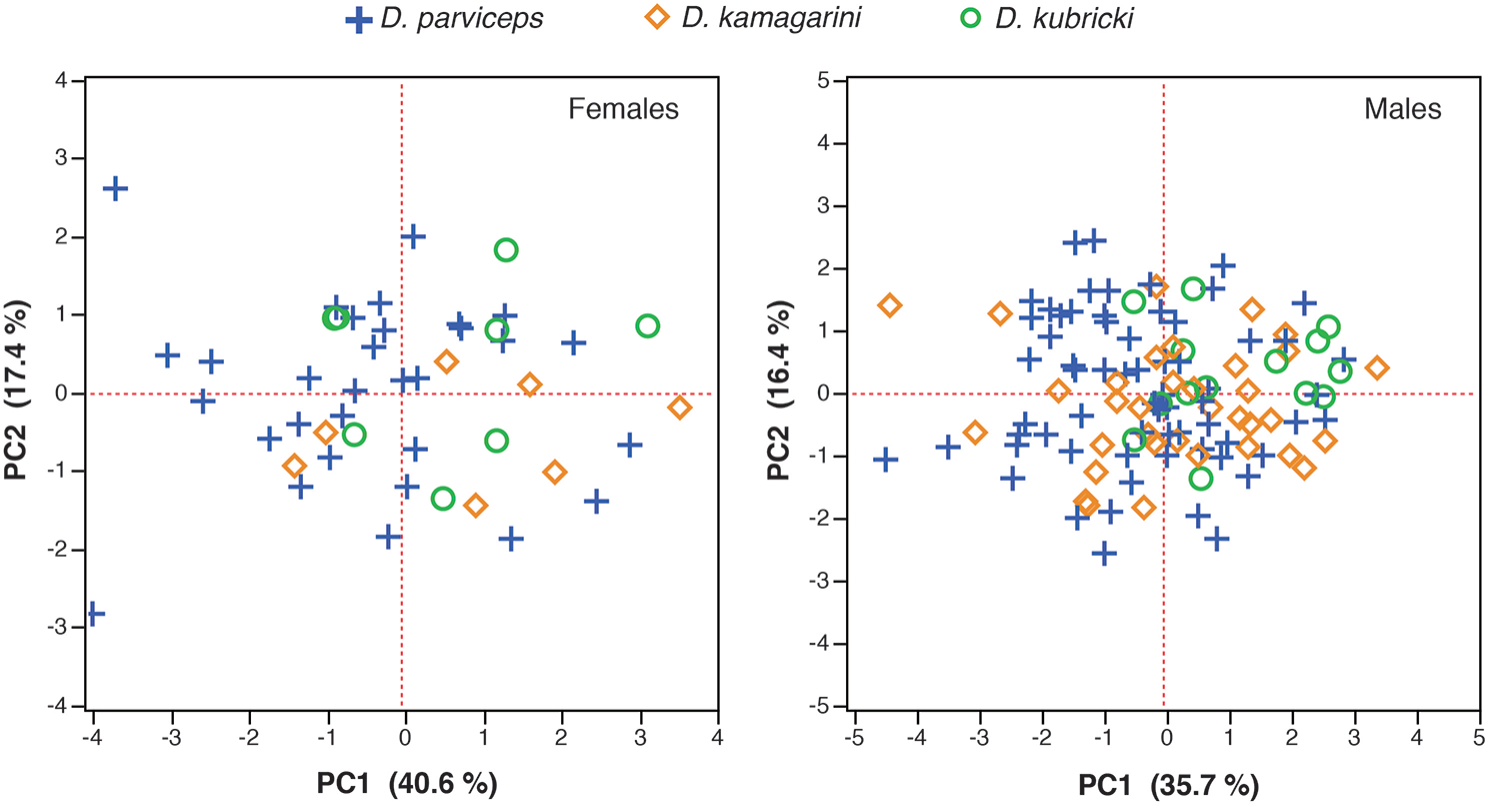
Principal components from analysis of seven size-corrected morphological variables of adults of *Dendropsophus parviceps* (Northern Clade), *D. kamagarini* sp. n. (Southern Clade), and *D. kubricki* sp. n. (Central Clade). The contribution of each axis to total variation is indicated in parenthesis.

Two components with eigenvalues > 1.0 were extracted from the PCA. Both PCs account for 52.1% of the total variation for males (Table 4). Principal Component I has high positive loadings for femur length and tibia length and PC II for head width and internarial distance (Table 4). The morphometric space shows high overlap between clades (Fig. 3).

**Table 4.** Character loadings and eigenvalues for Principal Components (PC) I–II. The analysis was based on seven size-corrected morphometric variables of adult *Dendropsophus parviceps* (Northern Clade), *D. kamagarini* sp. n. (Southern Clade), and *D. kubricki* sp. n. (Central Clade). Bold numbers indicate highest loadings.

Variables	PCA females		PCA males	
	PCI	PCII	PCI	PCII
Head width	**0.44**	-0.08	0.37	**0.44**
Head length	0.33	-**0.47**	0.35	0.08
Eye to nostril distance	0.24	**0.66**	0.34	0.14
Internarial distance	0.35	-0.12	0.13	**0.76**
Femur length	**0.44**	0.22	**0.51**	-0.16
Tibia length	**0.43**	0.32	**0.47**	-0.33
Foot length	0.39	-**0.41**	0.36	-0.26
Eingevalue	2.84	1.22	2.50	1.15
% of variation	40.6	17.4	35.7	16.4

Two PCs with eigenvalues > 1.0 explain the 58% of total variation among females (Table 4). The highest loadings for PC I were head width, femur length, and tibia length; PC II has high loadings for eye to nostril distance and is negatively correlated with head length and foot length (Table 4). As in the PCA for males, there is high overlap between clades in morphometric space (Fig. 3).

### Bioacoustic comparisons

**Figure 4.**
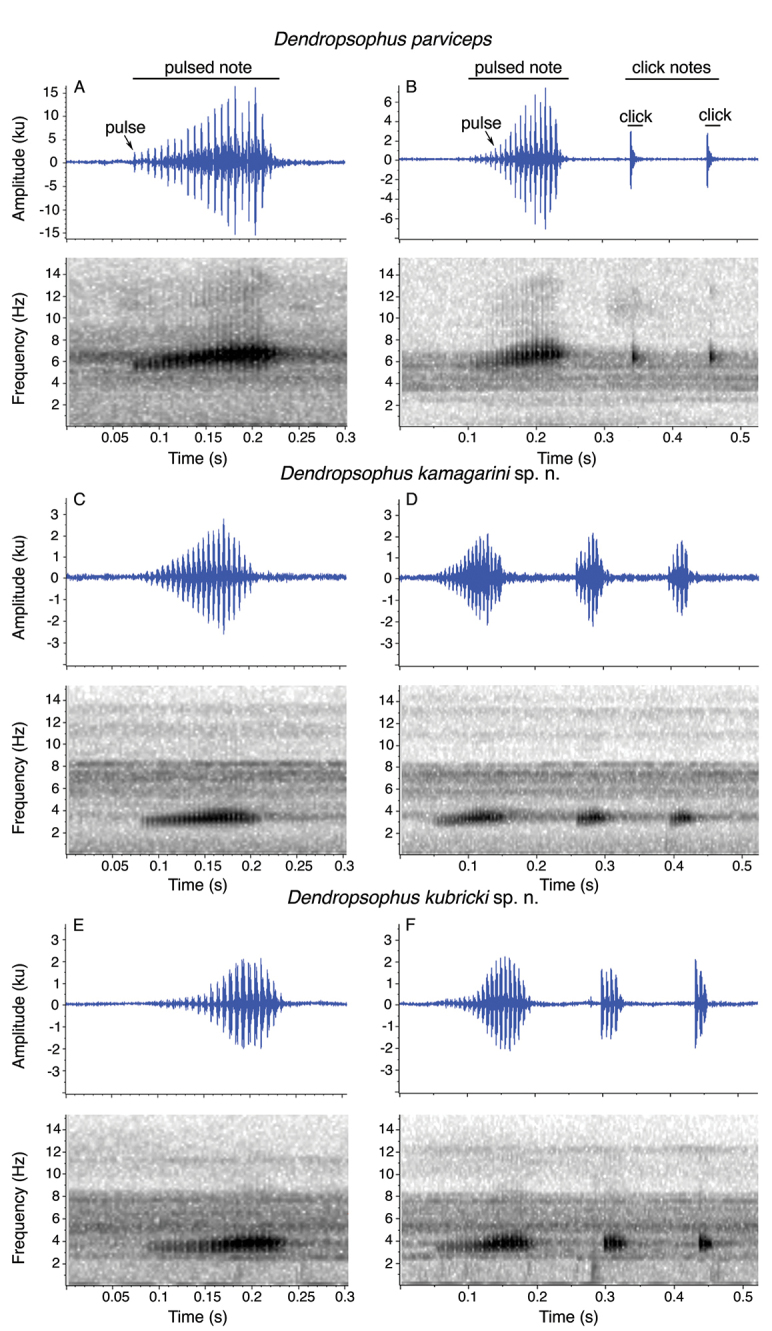
Advertisement calls of the *Dendropsophus parviceps* species complex. On each species, the oscillograms are shown above and spectrograms . *Dendropsophus parviceps* (Northern Clade): **A** Advertisement call from Sarayaku (QCAZ 52753) **B** Advertisement call + clicks from Canelos (QCAZ 52837). *Dendropsophus kamagarini* sp. n. (Southern Clade): **C** Advertisement call from Tambopata **D** Advertisement call + clicks from Tambopata. *Dendropsophus kubricki* sp. n. (Central Clade): **E** Advertisement call from Río Tapiche **F** Advertisement call + clicks from Río Tapiche. Calls from Peru lack specimen vouchers. A note-centered approach was used to define what is considered a call and a note (*sensu* Köhler et al. 2017).

The call of the *Dendropsophus parviceps* species complex consists of one pulsed trill (Fig. 4A, C, E). The pulsed trill is facultatively followed by one or more click notes (Fig. 4B, D, F). The pulsed trill appears to function as advertisement call because males produce these calls repeatedly and antiphonally. Acoustic parameters for the advertisement calls and click notes are shown in Table 5–7.

The dominant frequency of the advertisement call of the Northern Clade is higher (range 5081.8−6869.1 Hz) than that of the Southern Clade (range 3164.1−4306.6 Hz) and Central Clade (range 3542.2−4394.5 Hz). There are significant differences in dominant frequency for advertisement calls between the Northern Clade and the Southern Clade (Student’s t test, *t* = 13.68, df = 17, *p* < 0.001), and between the Northern Clade and the Central Clade (Student’s t test, *t* = 9.94, df = 13, *p* < 0.001). The number of pulses of the advertisement calls of Southern Clade is larger (12–32) than that of the Northern Clade (8–25; differences are significant: Student’s t test, *t* = -2.48, df = 17, *p* = 0.02).

**Figure 5.**
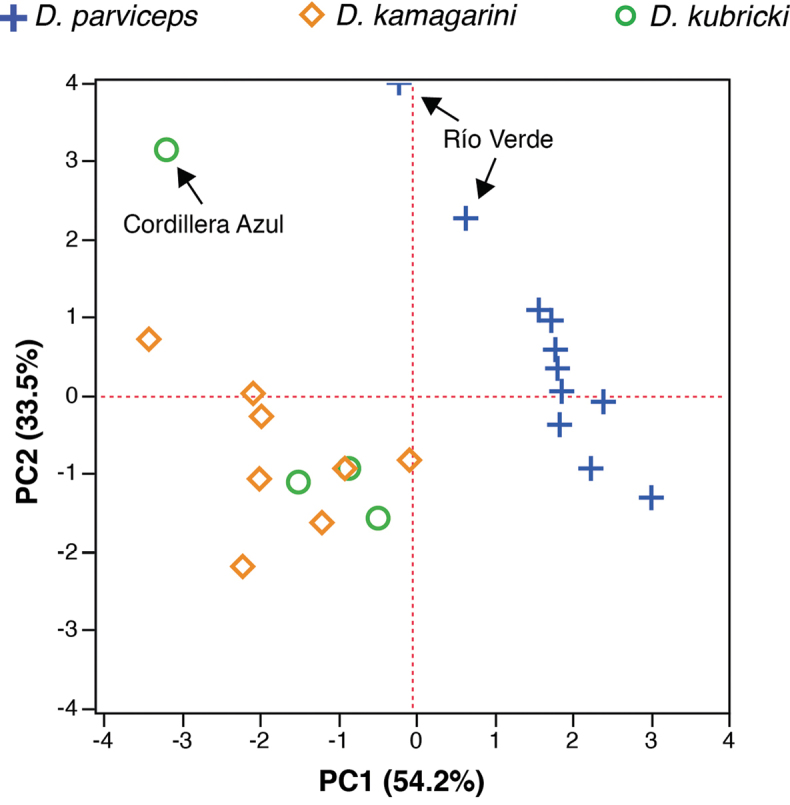
Principal components from analysis of seven acoustic variables of advertisement calls of *Dendropsophus parviceps* (Northern Clade), *D. kamagarini* sp. n. (Southern Clade), and *D. kubricki* sp. n. (Central Clade). The contribution of each principal component to explain total variation is indicated in parenthesis.

The PCA for advertisement calls shows that the Northern Clade is acoustically distinct from the Southern and Central clades (Fig. 5). Two components with eigenvalues > 1.0 account for 87.7% of the acoustic variation (Table 8). The highest loadings for PC I were dominant frequency, initial frequency, final frequency, and number of pulses; the highest loadings for PC II were note duration, rise time, and pulse rate. The northern clade differs from the southern and central clades mostly along PC I, which mainly represents variation in call frequency (Fig. 5).

### Species limits

The integrative analyses presented in this work show congruent differences in genetic, morphological, and bioacoustic characters that demonstrate the existence of three confirmed candidate species within “*Dendropsophus parviceps*”: Northern, Central, and Southern clades. Because the type locality of *Dendropsophus parviceps* is in Amazonian Ecuador (Sarayaku), we consider that the Northern Clade is *Dendropsophus parviceps* *sensu stricto.* This assignment is confirmed by the lack of tubercles in the eyelid of the holotype, a character state unique to the Northern Clade. Therefore, the two Peruvian species are new and we describe them in the following section.

### Systematic accounts

#### Dendropsophus parviceps

Taxon classificationAnimaliaAnuraHylidae

(Boulenger, 1882)

Figs 1 , 6 , 7A , 8

Hyla parviceps Boulenger, 1882: 393. Holotype BMNH 1947.2.13.51, an adult female from “Sarayacu”, Pastaza Province, Ecuador.
Hyla parviceps – Duellman and Crump 1974: 19; Duellman 1978: 156.
Dendropsophus parviceps – Faivovich et al. 2005: 93.

##### Diagnosis.

Throughout the species account, coloration refers to preserved specimens unless otherwise noted. *Dendropsophus parviceps* is characterized by: (1) small size, mean SVL 16.4 mm in males (range 14.3–18.7; *n* = 65), 22.5 mm in females (range 20.3–24.4; *n* = 30); (2) throat sexually dimorphic, dark flecks posteriorly in males vs. white blotch with two or three longitudinal stripes or without stripes posteriorly in females (Fig. 8); (3) snout truncate in dorsal and lateral views, slightly inclined posteroventrally in lateral view; (4) nostrils slightly prominent; (5) tympanum visible, concealed posterodorsally, tympanic membrane differentiated and annulus evident; (6) conical tubercles on upper eyelid absent; (7) thoracic fold absent; (8) ulnar tubercles and outer tarsal tubercles indistinct; (9) axillary membrane present; (10) skin on dorsal surfaces smooth with scattered small tubercles; skin on chest areolate; skin on belly, posterior surfaces of thighs, and subcloacal area coarsely areolate; skin on throat and other surfaces smooth; (11) dark brown markings on dorsum (Fig. 8); (12) thenar tubercle is distinct; (13) hand webbing formula II1^1/2^–2III2-–2-IV, feet webbing formula I1-−2-II1-−2-III1-–2IV2−1-V; (14) in life, dorsal surfaces brown, tan or grayish tan; (15) orange to amber blotch on the proximal ventral surface of shanks and under arms, from the axillae to near the elbow, in life (white to creamy white in preservative); (16) one suborbital white bar present both in life and preservative; (17) thighs are black to dark brown with two or three white spots on the anterodorsal surfaces both in life and preservative; (18) iris in life is creamy white to reddish brown with brow or dark brown reticulations.

##### Comparisons with other species.

*Dendropsophus parviceps* is most similar to *D. kamagarini* sp. n. and *D. kubricki* sp. n. The three species differ from other species of the *D. parviceps* group *sensu* Fouquet et al. 2015 (characters of other species of the group in parenthesis) by lacking dorsolateral light stripes [present in *D. bokermanni* (from Goin 1960) and in *D. brevifrons* (see Duellman and Crump 1974 and Read and Ron 2011)] and having, in life, an orange or amber blotch on the proximal ventral surface of shanks and under arms, from the axillae to near the elbow [absent in *D. bokermanni* (Goin 1960; Duellman and Crump 1974), in *D. brevifrons* (Duellman and Crump 1974), in *D. counani* (Fouquet et al. 2015), in *D. frosti* (Motta et al. 2012) and in *D. koechlini* (Duellman and Trueb 1989)]. *Dendropsophus parviceps* is also similar to *D. pauiniensis* (Heyer, 1977), but it can be distinguished by the presence of an orange or amber blotch on the proximal ventral surfaces of shanks in life (absent in *D. pauiniensis*).

*Dendropsophus parviceps*, *D. kamagarini* sp. n., and *D. kubricki* sp. n. further differ from species of the *D. parviceps* group (traits of other species of the *D. parviceps* group in parenthesis) as follows: from *D. koechlini* by having a white chest both in life and preserved [white with black flecks both in life and preserved (see Duellman and Trueb 1989)]; from *D. bokermanni*, *D. brevifrons*, *D. counani*, and *D. frosti* by having a mottled ventral coloration both in life and preserved [plain coloration both in life and preserved in *D. bokermanni* (from Goin 1960), in *D. brevifrons* (from Duellman and Crump 1974), in *D. counani* (from Fouquet et al. 2015), and in *D. frosti* (from Motta et al. 2012)]; from *D. bokermanni*, *D. brevifrons*, and *D. counani* by having a single suborbital bar [two suborbital bars (data of *D. bokermanni* and *D. brevifrons* from Duellman and Crump 1974, and of *D. counani* from Fouquet et al. 2015)] and two or three white spots on the anterior dorsal surfaces of the black thighs in life [cream or yellow spots in life (data of *D. bokermanni* and *D. brevifrons* from Duellman and Crump 1974, and of *D. counani* from Fouquet et al. 2015)]. The absence of canthal and rostral stripes also differentiates *D. parviceps*, *D. kamagarini* sp. n., and *D. kubricki* sp. n. from *D. bokermanni*, *D. brevifrons*, *D. frosti*, and *D. koechlini* [both stripes present in *D. bokermanni* and *D. brevifrons* (data of both species from Duellman and Crump 1974), canthal stripes in *D. frosti* (see Motta et al. 2012), and rostral stripes in *D. koechlini* (see Duellman and Trueb 1989)].

*Dendropsophus parviceps* differs from both new species by the absence of tubercles on the upper of eyelid (present). *Dendropsophus parviceps* also differs from *D. kamagarini* sp. n. and *D. kubricki* sp. n. by having translucent gray on the ventral surface of the thighs with dark brown flecks posteriorly in males, in life (black posteriorly in males, in life, in *D. kamagarini* sp. n. and in *D. kubricki* sp. n.).

##### Variation.

Morphometric variation is shown in Table 3. Variation in dorsal and ventral coloration of preserved specimens is depicted on Figure 8. Dorsal coloration  varies from brown (e.g., QCAZ 52026, 52816) to dark brown (e.g., QCAZ 52755, CORBIDI 1059), gray (e.g., QCAZ 52017), grayish tan (e.g., QCAZ 44441, 51230), or grayish brown (e.g., QCAZ 44736, 52026) with dark brown markings with varying shapes (Fig. 8). The specimens with gray, grayish tan, and grayish brown coloration have scattered iridophores. The dorsum is smooth (e.g., QCAZ 51108, 52755), but some specimens have scattered small tubercles (e.g., QCAZ 53181, CORBIDI 1046).

The chest is white to cream (Fig. 8) with throat and belly varying from creamy white (e.g., QCAZ 51230), grayish brown (e.g., QCAZ 48929, 52017) to dark brown (e.g., QCAZ 44440) with dark brown or black flecks. The subcloacal area is areolate, its coloration is white (e.g., QCAZ 44441, 48929), but in some specimens is dark brown (e.g., QCAZ 52755).

##### Color in life.

**Figure 6.**
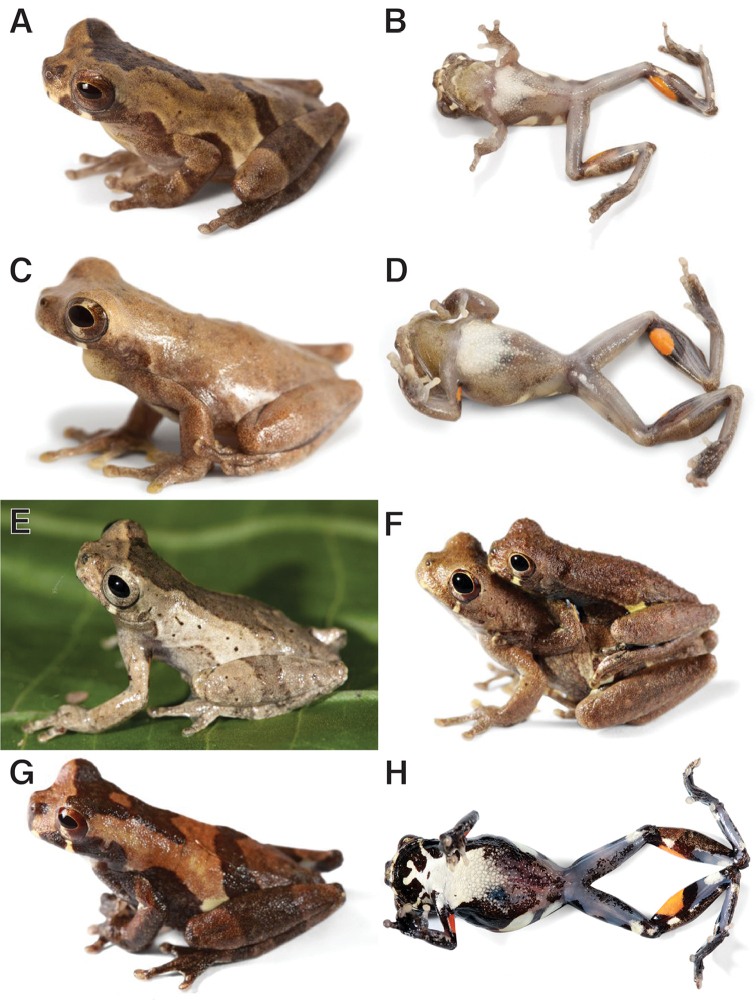
Dorsolateral and ventral views of *Dendropsophus parviceps* in life: **A, B** Adult male, from type locality Sarayaku, Pastaza, Ecuador (QCAZ 52752) **C, D** Adult male, from Canelos, Pastaza, Ecuador (QCAZ 52816) **E** Adult male, from Yasuní, Orellana, Ecuador (QCAZ 51073) **F** Amplectant pair from Nuevo Rocafuerte, Río Napo, Orellana, Ecuador (QCAZ 44773–74) **G, H** Adult female, from Chiroisla, Río Napo, Orellana, Ecuador (QCAZ 44440). Photographs by S. Ron.

Based on digital photographs (Fig. 6): dorsum varies from brown, tan, grayish tan to reddish brown, some individuals have few scattered dorsolateral dark brown flecks; dorsal markings are dark brown; flanks are white or creamy yellow with black or dark brown diagonal bars; dorsal surfaces of forelimbs and shanks have dark brown transversal bars; anterodorsally, thighs are black or dark brown with two or three white spots. The single suborbital bar is white. The venter is translucent gray mottled with black or dark brown; in some females venter is black; chest is white; in adult males, throat is olive tan mottled with dark brown flecks anteriorly and translucent gray posteriorly; in adult females, throat is grayish tan or olive brown, dark brown, or black anteriorly with a white blotch with stripes posteriorly; the ventral surfaces of the limbs are translucent gray or translucent white, thighs are mottled with dark brown posteriorly; there is one bright orange or amber blotch in ventral surface of shank next to the knee, and in the posterior arm, from the axillae to near the elbow. The iris is creamy white to reddish brown with brown or dark brown reticulations.

**Figure 7.**
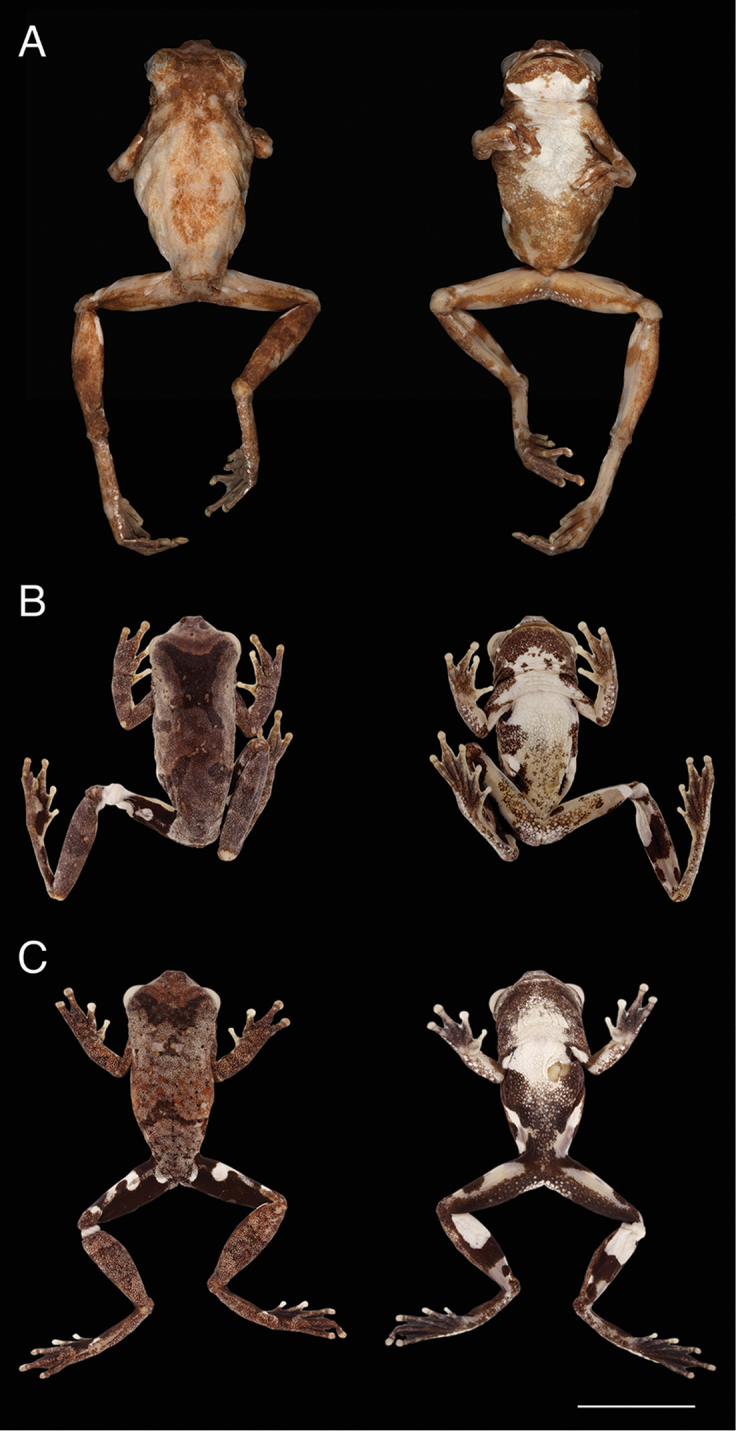
Dorsal and ventral views of the holotypes of the *Dendropsophus parviceps* species complex. **A** *Dendropsophus parviceps*, adult female, SVL = 26.55 mm (BMNH 1947.2.13.51) **B** *D. kamagarini* sp. n. adult male, SVL = 19.65 mm (CORBIDI 5246) **C** *D. kubricki* sp. n. adult male, SVL = 19.05 mm (CORBIDI 15778). Scale bar 10 mm.

**Figure 8.**
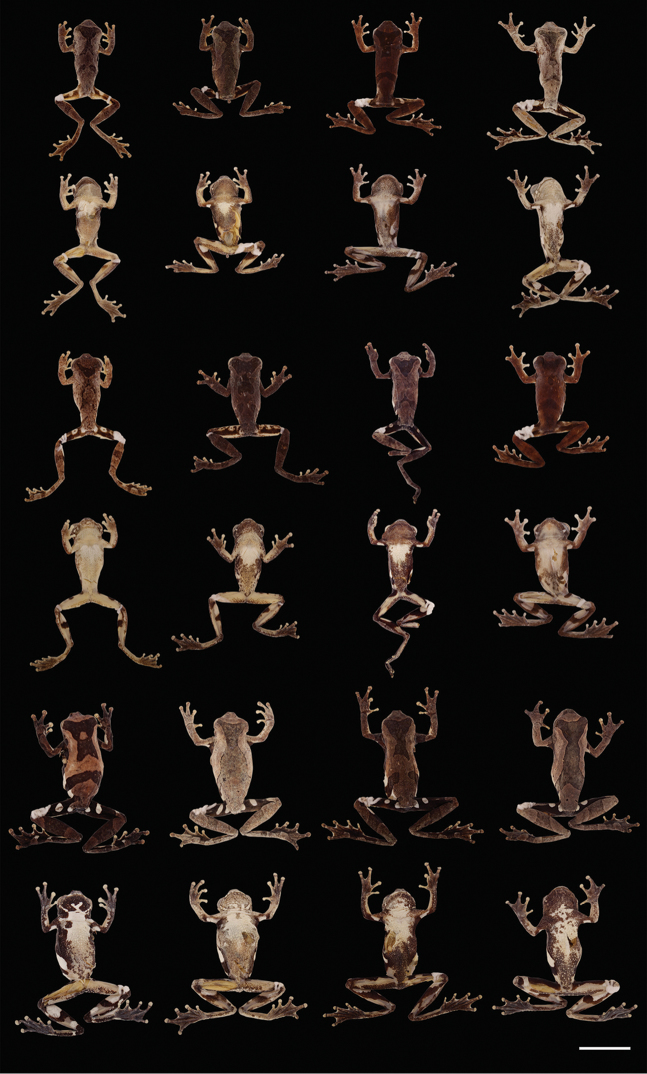
Adults of *Dendropsophus parviceps* showing variation in dorsal and ventral coloration of preserved specimens. From left to right, first and second rows: QCAZ 52017, 52026, 52755, 51230 (males); third and fourth rows: CORBIDI 1040, 1059, QCAZ 48929, 52816 (males); fifth and sixth rows: QCAZ 44440–41, 27983, 44736 (females). See Appendix 1 for locality data. Scale bar 10 mm.

##### Calls

(Fig. 4A−B). Descriptive statistics of acoustic variables are provided in Table 5. Calls from ten individuals were analyzed. Three individuals (two of them unvouchered specimens and QCAZ 52753) were recorded at the type locality, Sarayaku, Pastaza Province, at night, on 6 April 2012 (QCAZ 52753 was recorded at 01:00h, temperature 22.4°C). Three individuals (QCAZ 52820, 52837 and one individual not collected) were recorded at Canelos, Pastaza Province, on 11 April 2012 (QCAZ 52820 recorded at 01:00h, 23.4°C). Two individuals (QCAZ 52017, 52918) were recorded at Río Verde, Tungurahua Province, on 19 September 2011 (QCAZ 52018, air temperature = 15.6°C). Finally, two individuals, not collected, were recorded at PUCE’s Yasuní Research Station, Orellana Province, on 1 June 2011. We obtained one recording (unvouchered specimen) from the sound archives of Museo de Zoología, Pontificia Universidad Católica del Ecuador, made by Morley Read, at Pompeya-Iro road, km. 38, Yasuní National Park, Orellana Province.

**Table 5.** Acoustic parameters of *Dendropsophus parviceps* (Northern Clade). Mean ± SD is given with range below. Sample sizes are number of calls. All frequencies are in Hz and durations in s.

	*Dendropsophus parviceps*				
	Sarayaku (*n* = 3)	Canelos (*n* = 3)	Río Verde (*n* = 2)	Yasuní (*n* = 3)	Combined (*n* = 11)
Advertisement call duration	0.14 ± 0.03 (0.06−0.18)	0.13 ± 0.02 (0.10−0.17)	0.19 ± 0.04 (0.11−0.24)	0.11 ± 0.03 (0.06−0.18)	0.14 ± 0.04 (0.06−0.24)
Advertisement call dominant frequency	6523.1 ± 184.18 (6115.4−6836.8)	6454.1 ± 146.63 (6169.3−6686.1)	5364.7 ± 167 (5081.8−5824.7)	6490.4 ± 300.7 (5953.9−6869.1)	6278.8 ± 503.75 (5081.8−6869.1)
Advertisement call initial frequency	5997.4 ± 223.5 (5674−6546.1)	6020.5 ± 253.51 (5630.9−6352.3)	5074.7 ± 260.71 (4758.8−5835.5)	6130.3 ± 227.74 (5717.1−6729.1)	5870.1 ± 459.02 (4758.8−6729.1)
Advertisement call final frequency	6602.4 ± 202.04 (6126.2−6836.8)	6565.7 ± 186.92 (6147.7−6750.7)	5419.8 ± 178.33 (5103.4−5835.5)	6567.64 ± 261.07 (6007.8−6966)	6356.5 ± 510 (5103.4−6966)
Advertisement call rise time	0.07 ± 0.01 (0.03−0.09)	0.07 ± 0.01 (0.05−0.01)	0.10 ± 0.02 (0.05−0.12)	0.06 ± 0.01 (0.04−0.09)	0.07 ± 0.02 (0.03−0.12)
Number of pulses of advertisement call	17.53 ± 3.22 (8−22)	17 ± 1.10 (16−19)	16.61 ± 3.53 (8−20)	14.2 ± 4.45 (9−25)	16.1 ± 3.87 (8−25)
Advertisement call pulse rate	126.76 ± 10.6 (97.83−146.34)	133.43 ± 13.60 (111.76−152.38)	86.1 ± 11.79 (65.57−125.9)	126.57 ± 19.35 (77.92−171.88)	119.61 ± 22.2 (65.57−171.88)
Call duration	0.45 ± 0.21 (0.27−0.90)	0.44 ± 0.20 (0.26−0.77)	0.61 ± 0.30 (0.37−1.22)	0.72 ± 0.4 (0.26−2.12)	0.6 ± 0.3 (0.26−2.12)
Inter note interval	0.08 ± 0.021 (0.03−0.10)	0.07 ± 0.02 (0.05−0.11)	0.12 ± 0.017 (0.10−0.15)	0.08 ± 0.012 (0.06−0.12)	0.09 ± 0.02 (0.03−0.15)
Click note duration	0.05 ± 0.014 (0.03−0.076)	0.052 ± 0.015 (0.028−0.075)	0.051 ± 0.015 (0.03−0.082)	0.042 ± 0.011 (0.028−0.078)	0.05 ± 0.013 (0.028−0.082)
Click note dominant frequency	6334.2 ± 151.8 (5964.7−6567.6)	6471.9 ± 194.80 (6190.8−6761.4)	5284.8 ± 109.5 (4866.5−5415.6)	6544.9 ± 207.7 (5997−6922.9)	6334.6 ± 460.3 (4866.5−6922.9)
Number of pulses of click note	2.2 ± 1.3 (1−5)	3.3 ± 1.7 (1−6)	1.65 ± 0.92 (1−5)	2.1 ± 0.84 (1−4)	2.1 ± 1.08 (1−6)
Click note rise time	0.024 ± 0.007 (0.015−0.038)	0.024 ± 0.008 (0.014−0.038)	0.025 ± 0.008 (0.015−0.042)	0.021 ± 0.006 (0.013−0.039)	0.023 ± 0.007 (0.013−0.042)
Click note pulse rate	47.96 ± 31.29 (14.08−138.89)	59.10 ± 22.22 (20.83−100)	31.37 ± 9.95 (18.52−60.98)	48.54 ± 15.3 (20−103.45)	46.89 ± 20.06 (14.08−138.89)
Inter click notes interval	0.064 ±0.016 (0.031−0.088)	0.066 ± 0.019 (0.017−0.088)	0.12 ± 0.03 (0.08−0.18)	0.075 ± 0.012 (0.038−0.098)	0.08 ± 0.023 (0.017−0.18)

The advertisement call is a pulsed note (Fig. 4A−B). The amplitude increases gradually at the beginning and falls sharply towards the end. The advertisement call may be emitted alone or followed by one or more click notes. However, the click notes occasionally are emitted alone. The click notes may be non-pulsed or pulsed.

*Call comparisons between populations*. The advertisement calls from Río Verde separate along PC II from the calls of other populations (Fig. 5; Table 8). Mean dominant frequency is 5364.7 Hz (SD = 167) at Río Verde and 6498.1 Hz (SD = 239) at the other populations. Mean pulse rate is 86.1 pulses/s (SD = 11.79) at Río Verde and 127.6 pulses/s (SD = 15.5) at others. Mean rise time is 0.1 (SD = 0.02) and mean advertisement call duration is 0.19 s (SD = 0.04) at Río Verde, while mean rise time is 0.06 (SD = 0.01) and mean advertisement call duration is 0.12 s (SD = 0.03) at the other populations.

##### Distribution and ecology.

**Figure 9.**
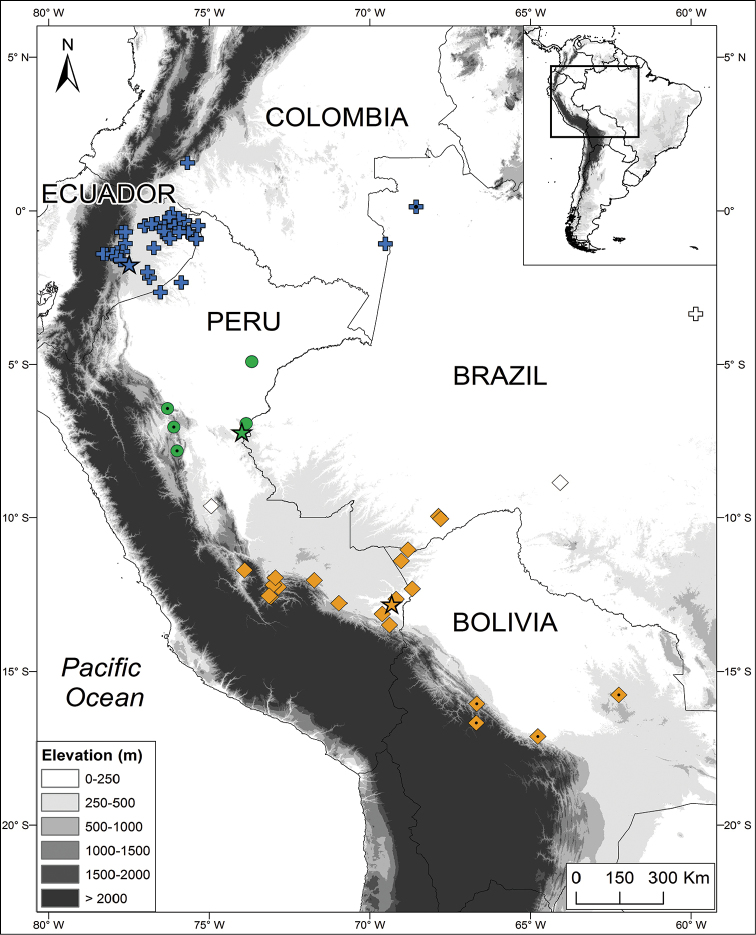
Distribution of *Dendropsophus parviceps* species complex. *Dendropsophus parviceps* (Northern Clade, blue crosses), *D. kubricki* sp. n. (Central Clade, green circles), *D. kamagarini* sp. n. (Southern Clade, orange rhombi). Stars = type locality, figures with a small black dot at the center = referred specimens, and hollow figures = unconfirmed records.

*Dendropsophus parviceps* is known from 39 localities in the Ecuadorian Amazon basin (Napo, Orellana, Pastaza, Sucumbíos, and Tungurahua provinces; Fig. 9), few localities in the Peruvian Amazon basin at northwest Loreto (Andoas and San Jacinto; Fig. 9), the Colombian Amazon (Río Apaporis, Vaupés Department, and Ceilán, Caquetá Department; Cochran and Goin 1970; Fig. 9), and northern Brazil (“Taracuá” [= Taracuacá], Río Uaupés, Amazonas State; Melin 1941; see Remarks section). Elevation range is 151 m (Andoas) to 1600 m above sea level (Río Verde). Our Colombian records are unverified and are based on Cochran and Goin (1970) who examined three specimens (MLS 54 and MCZ 28058–59) and explicitly mention the absence of tubercles on the upper eyelids. Moreover, the SVL for a gravid female from Ceilan (MLS 54, 21.8 mm) falls outside the known size range of *D. kubricki* sp. n. and *D. kamagarini* sp. n. (Table 3). Ecuadorian localities from Sucumbíos province are close to the Colombian border further suggesting the presence of *D. parviceps* in Colombia. In addition, there is an unconfirmed register of *D. parviceps* from Ramal do Purupuru, km 34 on the BR-319 highway (3.3535°S, 59.8557°W, 35 m, Amazonas State, Brazil; Fig. 9).

*Dendropsophus parviceps* inhabits Amazonian lower montane forest, Amazonian foothill forest, and Amazonian evergreen lowland rainforest (habitat types based on Ron et al. 2017). *Dendropsophus parviceps* is an opportunistic breeder and can be found in primary and secondary forest, temporary ponds, flooded areas, swamps, and artificial open areas. Calling activity starts at dusk (17–18h), but it is mainly nocturnal. According to Lynch (2005), *D. parviceps* is a canopy species that visits the lower forest strata for breeding.

##### Conservation status.

Its extent of occurrence is 256,944 km^2^. There is habitat degradation and fragmentation within its distribution as result of human activities, especially cattle rising, agriculture, and oil exploitation. Its presence in artificial open areas suggests that is tolerant of at least some level of habitat modification (Azevedo-Ramos et al. 2004). Its distribution range is large and includes extensive undisturbed areas (Ministerio de Ambiente Ecuador 2013). Therefore, we propose that *D. parviceps* should be assigned to the Least Concern category, following IUCN (2001) criteria.

##### Remarks.

The advertisement call from Río Verde differs from other population calls (Fig. 5; Table 5). However, low genetic and morphological differences between Río Verde and the other populations indicate that they are conspecific. The Brazilian record from Tarauacá, Río Uaupés (Amazonas State) is based on Melin (1941) who reported a juvenile specimen with SVL = 21 mm. This specimen could be an adult male because the throat is mottled with brown, characteristic of all adult males of *Dendropsophus parviceps*. Nevertheless, the SVL of the male from Taracuá falls above the range of variation of males of *D. parviceps* (14.3−18.7 mm) and it has a thoracic fold (fold absent in *D. parviceps*; see above in Diagnosis section). Therefore, the record from Tarauacá requires verification.

The holotype has SVL = 26.5 mm (adult female; Fig. 7A). This value is above the range of variation of females of *D. parviceps* reported in Table 3 (20.3−24.4 mm). To confirm that the holotype falls within the range of variation of *D. parviceps* from Ecuador, we measured the SVL of the largest adult females from the QCAZ collection. We found three specimens with size close to the holotype: QCAZ 4340 (SVL = 26.13 mm) from La Selva (Sucumbíos Province), QCAZ 27028 (SVL = 26.03 mm) from Ahuano (Napo Province), and QCAZ 59772 (SVL 26.26 mm) from Comunidad Zarentza (Pastaza Province; Appendix 1). Although the holotype is the largest specimen known for *D. parviceps*, other specimens are smaller by just ~1% of SVL. Other characteristics of the external morphology of the holotype fall within the known variation of the Ecuadorian populations confirming that they are conspecific (Figs 7A, 8).

#### Dendropsophus kamagarini sp. n.

Taxon classificationAnimaliaAnuraHylidae

http://zoobank.org/85BACA9D-07C6-4C1C-A818-B83DDD1510CA

**Figure 10.**
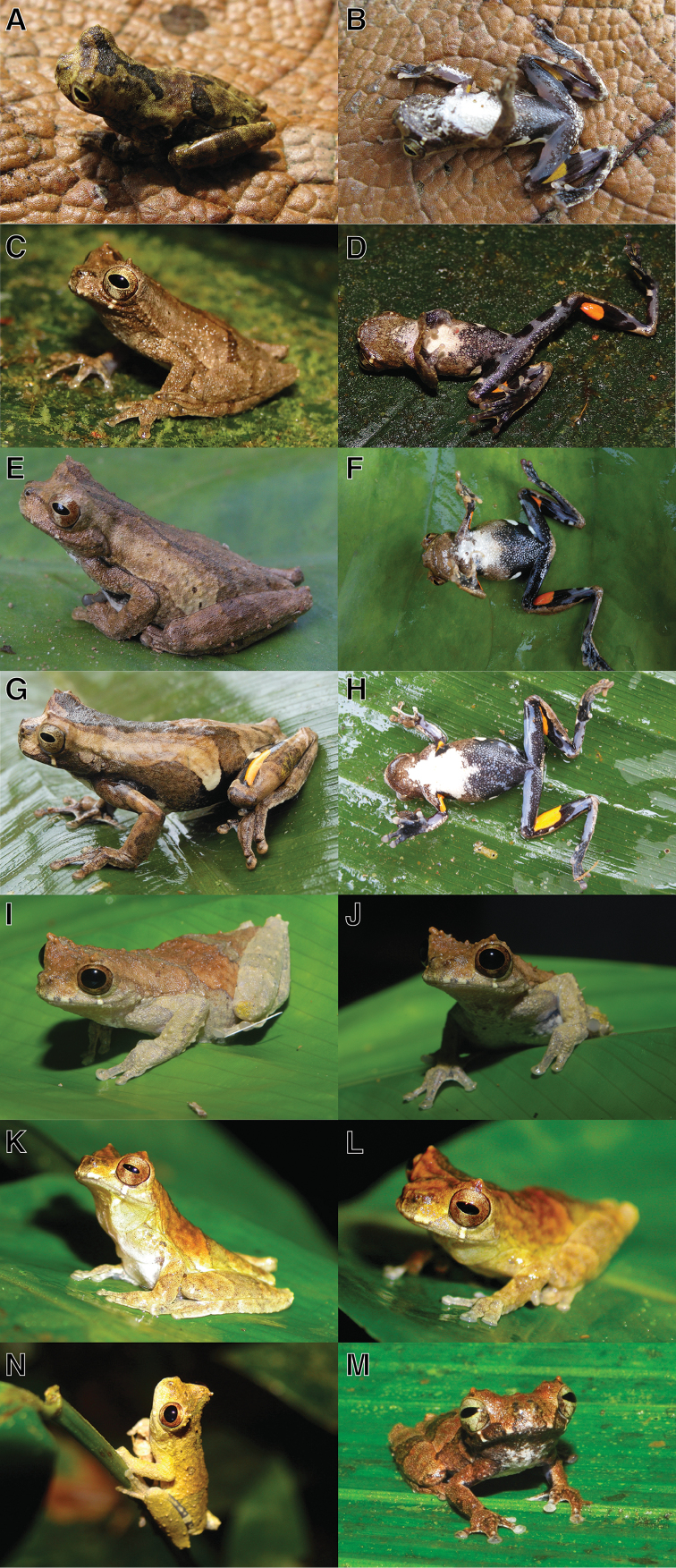
Dorsolateral and ventral views of *Dendropsophus kamagarini* sp. n. in life: **A, B** Adult male, from La Habana, Tambopata, Peru (CORBIDI 5259) **C, D** Adult male, from Bahuaja, Puno, Peru (CORBIDI 13148) **E–H** Adult females, from Pagoreni norte, La Convención, Peru **E, F** not collected. Dorsolateral and ventral views of *Dendropsophus kamagarini* sp. n. in life: **G, H** (CORBIDI 10018) **I, J** Adult male, from Tahuamanu, Nicolás Suárez, Bolivia (11.4074°S, 69.0180°W, 260 m, not collected) **K, L** Adult male, from El Negro, Manuripi, Bolivia (12.3134°S, 68.6689°W, 187 m, not collected) **N** Adult male, from Rio Branco, Acre, Brazil (10.0387°S, 67.7957°W, 160 m, not collected) **M** Adult male, from Rio Madeira, Rondônia, Brazil (8.8482°S, 64.0689°W, 110 m, not collected). Photos **A, B, E–H** by V. Duran, **C, D** by P. J. Venegas **I–L** by A. Muñoz, **N** by P.R. Melo-Sampaio, and **M** by A.P. Lima.

**Figure 11.**
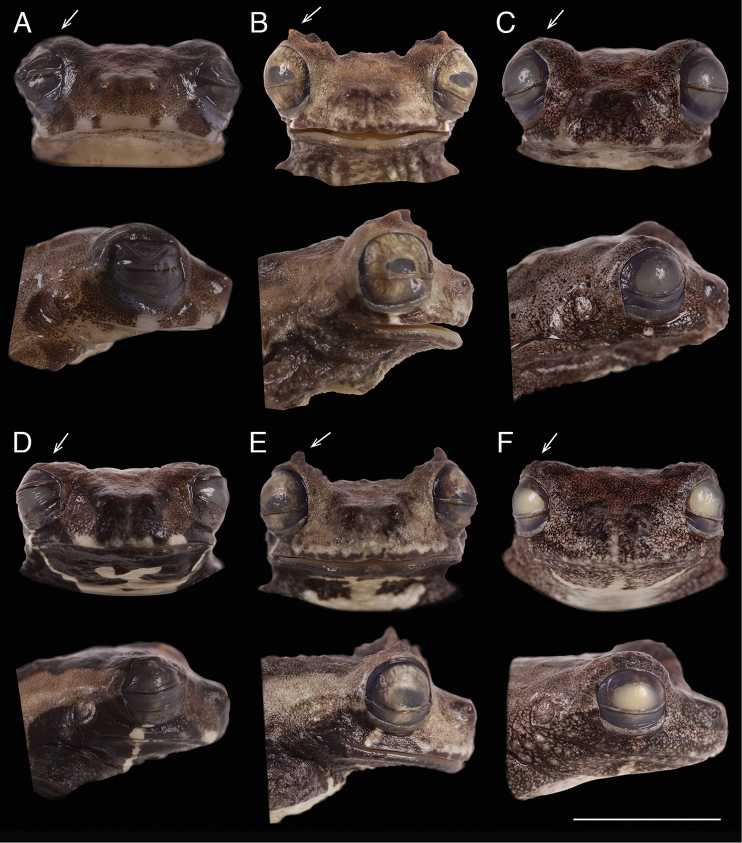
Adults of *Dendropsophus kamagarini* sp. n. showing variation in dorsal and ventral coloration of preserved specimens. From left to right, first and second rows: CORBIDI 8232, 8229, 8151, 8234 (males); third and fourth rows: CORBIDI 5480, 10019, 5471, 8305 (males); fifth and sixth rows: CORBIDI 8463, 10018, 6694 (females). See Appendix 1 for locality data. Scale bar 10 mm.

Figs 1 , 7B , 10 , 11

##### Holotype.

CORBIDI 5246, an adult male from Peru, Madre de Dios Department, Tambopata Province, Inotawa Lodge (12.8092°S, 69.3182°W), 192 m above sea level, collected on 9 October 2009 by P. J. Venegas.

##### Paratypes.

CORBIDI 5259, an adult male from Peru, Madre de Dios Department, Tambopata Province, La Habana (12.6537°S, 69.1796°W), 192 m above sea level, collected on 18 October 2009 by V. Durán and M. Cuyos. Thirty-three adult males and seven adult females from Peru, Cusco Department, La Convención Province: Comunidad Ochigoteni (12.5758°S, 73.0900°W), 1696 m above sea level, CORBIDI 5392, adult female, collected on 19 October 2009 by G. Chávez; Pongo de Mainique (12.2581°S, 72.8425°W), 670 m above sea level, CORBIDI 5471, 5473, 5480, 5484, adult males, collected on 23 April 2010 by G. Chávez; Megantoni (12.2581°S, 72.8425°W), 670 m above sea level, CORBIDI 6659, 6664, 6679, 6685, 6687–88, 6698, adult males, CORBIDI 6692, 6694, adult females; Comunidad Nativa Chokoriari (11.9569°S; 72.9409°W), 434 m above sea level, CORBIDI 8067–68, 8070, adult males, CORBIDI 8069, adult female, collected on 8 December 2010 by D. Vásquez; Comunidad Nativa Poyentimari (12.1885°S, 73.0009°W), 725 m above sea level, CORBIDI 8150–51, 8153, 8228−36, 8285–86, 8305, 8476, adult males, CORBIDI 8152, 8463, adult females, collected on 28 November 2010 by G. Chávez; Puyantimari (12.1861°S, 73.0004°W), 710 m above sea level, CORBIDI 9762, adult male, collected on 8 September 2011 by D. Vásquez and K. García; Pagoreni norte (11.7115°S, 73.8967°W), 402 m above sea level, CORBIDI 10018, adult female, CORBIDI 10019, adult male, collected on 22 November 2011 by V. Durán; Palmeiras-Alto Shimá (12.5453°S, 73.1350°W), 1500 m above sea level, CORBIDI 10585, adult female, collected on 7 February 2012 by G. Chávez and D. Vásquez; Chokoriari (11.9569°S, 72.9409°W), 413 m above sea level, CORBIDI 10628, adult male, collected on 19 February 2012 by G. Chávez and D. Vásquez.

##### Referred specimens.

Two adults from Bolivia, Cochabamba Department, Ayopaya Province: confluence of the Altamachi and Ipiri rivers (16.0543°S, 66.6667°W), 600 m above sea level, MHNC-A 427, 429, collected on 15 September 2004 by A. Muñoz and G. Rey. An adult from Bolivia, Cochabamba Department, Carrasco Province: Valle del Sacta (17.118°S, 64.767°W), 230 m above sea level, MHNC-A 2116, collected on 18 April 2014 by G. Callapa, A. Muñoz, D. Ercken, S. Barron, and M. Careaga.

##### Etymology.

The specific name *kamagarini* is a noun derived from the Matsigenka language, which means demon or devil (Snell et al. 2011). The Matsigenka language is spoken by the Matsigenka people who inhabit the highlands and lowlands of southeastern Peru, in the departments of Cusco and Madre de Dios. Judeo-Christian religions depict the demon as a human figure with horns. The species name is in allusion to the prominent horn-like tubercles on the upper eyelid of *D. kamagarini*.

##### Diagnosis.

**Figure 12.**
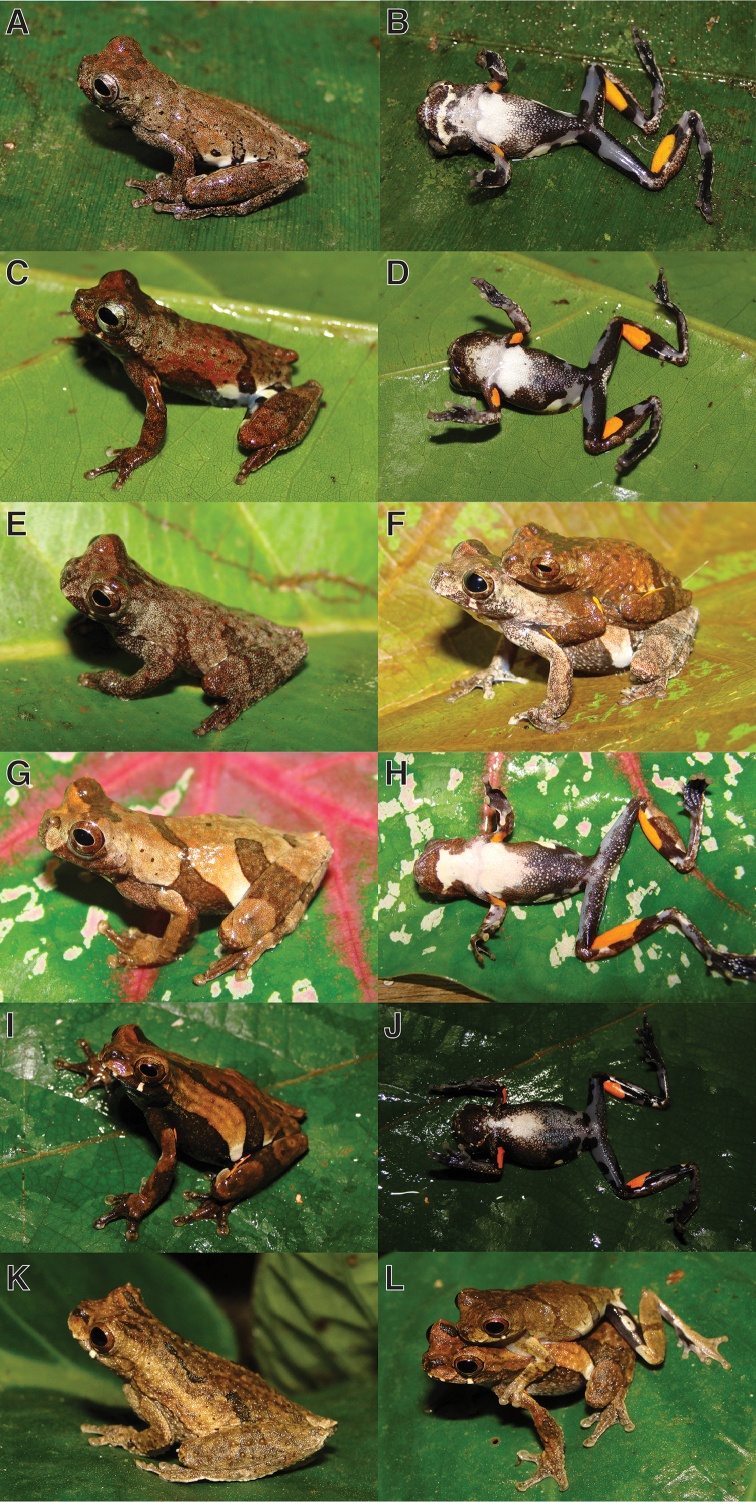
Frontal and lateral views of the head of adults of *Dendropsophus parviceps* species complex. *Dendropsophus parviceps*: **A** Male (QCAZ 52752) and **D** Female (QCAZ 44440) without tubercles (indicated by arrow); *D. kamagarini*: **B** Male (CORBIDI 8151) and **E** Female (CORBIDI 8152) with a conspicuous tubercle (arrow); *D. kubricki*: **C** Male (CORBIDI 15776) and **F** Female (CORBIDI 15785) with small tubercles (arrow). Note the snout shape in lateral view, truncate to slightly inclined posteroventrally in *D. parviceps*, truncate in *D. kamagarini*, and rounded and inclined posteroventrally in *D. kubricki*. Scale bar 5 mm.

Throughout the species description, coloration refers to preserved specimens unless otherwise noted. The new species is assigned to the genus *Dendropsophus* based on our phylogenetic results (Fig. 1) and the overall similarity with *D. parviceps* and other species of the genus (Figs 10–11). *Dendropsophus kamagarini* is a medium-sized species, relative to other species in the *D. parviceps* group and is characterized by the following combination of traits: (1) size sexually dimorphic; mean SVL 19.9 mm in males (range 17.6–22.7; *n* = 35), 26.1 mm in females (range 24.0–28.1; *n* = 7); (2) throat brown mottled with white flecks posteriorly in males vs. white blotch with flecks or with stripes posteriorly in females (Fig. 11); (3) snout is short and truncate in dorsal and lateral views; (4) nostrils slightly protuberant; (5) tympanum visible, tympanic membrane non-differentiated, annulus distinct; (6) one prominent conical tubercle on the distal edge of the upper eyelid; (7) thoracic fold indistinct to barely evident; (8) ulnar tubercles and outer tarsal tubercles distinct; (9) axillary membrane present; (10) skin on dorsal surfaces smooth with scattered tubercles; skin on chest, belly, posterior surfaces of thighs, and subcloacal area coarsely areolate; skin on throat grooved with scattered tubercles; (11) dark brown markings on dorsum (Fig. 11); (12) thenar tubercle distinct; (13) hand webbing formula II1-–2^+^III1-–1-IV, feet webbing formula I1^1/2^–2^+^II1-–1III1-–2-IV2–1V; (14) in life, dorsum tan, brown or reddish brown; (15) orange to amber blotch on the proximal ventral surface of shanks and under arms, from the axillae to near the elbow, in life (white to creamy white in preserved); (16) one suborbital white bar present both in life and preserved; (17) thighs black to dark brown with two or three spots on the anterodorsal surfaces both in life and preserved; (18) iris in life creamy white with brown to reddish brown reticulations and a cream ring around pupil.

##### Comparisons with other species.

*Dendropsophus kamagarini* is most similar to *D. parviceps* and *D. kubricki* sp. n. It can be distinguished from *D. parviceps* by having a prominent conical tubercle on the distal edge of the upper eyelid (tubercle absent in *D. parviceps*; Fig. 12) and a blunt and short snout in lateral view (slightly inclined posteroventrally in profile in *D. parviceps*; Fig. 12). *Dendropsophus kamagarini* is larger than *D. parviceps* (Fig. 2; see Morphological comparisons) and has a throat with white flecks posteriorly in males both in life and preserved (dark flecks posteriorly in males both in life and preserved in *D. parviceps*). Advertisement calls of *D. kamagarini* also have lower dominant frequency than those of *D. parviceps* (Fig. 4A–D; see Bioacoustic comparisons) and more pulses in the advertisement call (less pulses in *D. parviceps*; Fig. 4A–D; see Bioacoustic comparisons). *Dendropsophus kamagarini* differs from *D. kubricki* sp. n. by having a prominent conical tubercle on the distal edge of the upper eyelid (scattered low tubercles in *D. kubricki*; Fig. 12).

*Dendropsophus kamagarini* differs from other species of the *D. parviceps* group (*sensu* Fouquet et al. 2015) by having an orange or amber blotch on the proximal ventral surface of shanks and arms in life and a prominent conical tubercle on the distal edge of the upper eyelid (orange blotches and tubercle absent in *D. bokermanni* [Goin 1960; Duellman and Crump 1974], in *D. brevifrons* [Duellman and Crump 1974], in *D. counani* [Fouquet et al. 2015], in *D. frosti* [Motta et al. 2012] and in *D. koechlini* [Duellman and Trueb 1989]). *Dendropsophus kamagarini* also resembles *D. pauiniensis*, but it can be distinguished by the presence of an orange or amber blotch on the proximal ventral surface of shanks and a prominent conical tubercle on the distal edge of the upper eyelid (blotch and tubercle are absent in *D. pauiniensis*; Heyer 1977).

##### Description of holotype.

Adult male (Fig. 7B), SVL 19.6 mm. Head as wide as body, wider than long, widest below eyes; snout truncate and short in dorsal view, slightly inclined posteroventrally in lateral view; loreal region flat; lips thin; internarial region slightly concave; nostrils slightly protuberant dorsally and laterally; interorbital area flat; tympanum rounded distinct, tympanic annulus evident, tympanic membrane non-differentiated, supratympanic fold thin, restricted to upper edge of tympanum. Arms slender, not hypertrophied; axillary membrane extending to one third of upper arm; ulnar fold distinct, low ulnar tubercles present; fingers short, bearing small, round discs; relative length of fingers I < II < IV < III; subarticular tubercles small, round on fingers I and II, bifid on finger III, and divided on finger IV; supernumerary tubercles small, slightly evident; thenar tubercle distinct; palmar tubercle flat, round; webbing basal between fingers I and II; webbing formula of fingers II1-–2III1^1/2^–1-IV. Hindlimbs long, slender; tarsal fold absent, outer tarsal tubercles present, low; calcar and heel tubercles absent; toes bearing round discs, smaller than those of fingers; relative length of toes I < II < III < V < IV; subarticular tubercles small, round; supernumerary tubercles indistinct; inner metatarsal tubercle small, flat, elliptical; outer metatarsal tubercle absent; webbing formula of toes I1^1/2^–1-II1-–1III1-–2IV2–1-V. Skin on dorsum, dorsal surfaces of limbs, flanks, and groin smooth; skin on head smooth with scattered tubercles and one prominent conical tubercle on the distal edge of the upper eyelid; skin on venter, chest, posterior surfaces of thighs, and subcloacal area coarsely areolate; skin on throat and ventral surfaces of limbs smooth. Cloacal opening directed posteriorly at upper level of thighs; cloacal sheath short; cloacal folds and tubercles absent. Tongue cordiform, barely free posteriorly; dentigerous process of vomers small, prominent, narrowly separated, each bearing three and two vomerine teeth (left/right), positioned obliquely to choanae; choanae small, rounded; vocal slits long, extending from midlateral base of tongue to angle of jaws; vocal sac single, median, subgular.

##### Color of holotype in preservative.

Figure 7B. Dorsal surfaces of head, body, and limbs brown, grayish tan dorsolaterally with dark brown markings on dorsum consisting of median blotch anteriorly, transverse bars posteriorly; dark brown broad transverse bars on the forelimbs and shanks; anterodorsal surfaces of thighs black with three white spots; white suborbital bar. Ventral surface of belly white anteriorly, creamy mottled posteriorly with dark brown scattered flecks; chest white, throat brown anteriorly and white with brown flecks posteriorly; ventral surfaces of limbs creamy.

##### Measurements of holotype (in mm).

SVL 19.6, HW 6.3, HL 5.9, END 2.1, IN 2.0, FL 10.4, TL 10.7, FL 8.6.

##### Variation.

Morphometric variation in the paratype series is given in Table 3. Variation in dorsal and ventral coloration of preserved specimens is shown in Figure 11.  Dorsal coloration in preservative varies from gray (e.g., CORBIDI 8305, 10019) to grayish tan (e.g., CORBIDI 8232, 8234), brown (e.g., CORBIDI 5471, 5480), dark brown (e.g., CORBIDI 6694, 8229), reddish brown (e.g., CORBIDI 8463) or creamy tan (e.g., CORBIDI 8151) with dark brown markings (Fig. 11); some specimens have a blotch in occipital region, a blotch in scapular region, and a transverse blotch extending onto flanks in sacral region (e.g., CORBIDI 8234) or two “)(” shaped stripes beginning on the upper eyelids, extending onto the flanks, and reaching the sacral region; an indistinct creamy middorsal line extends from the occipital region to the sacral region (e.g., CORBIDI 6694, 8151); some specimens have brown, creamy or grayish tan stripes around the dark brown markings (e.g., CORBIDI 10018). The dorsum has scattered tubercles, mainly on head and upper eyelid (e.g., CORBIDI 5471, 6694), but in some specimens the dorsum is smooth (e.g., CORBIDI 8069, 10018). The prominent conical tubercle on the upper eyelid in live individuals becomes smaller in preserved specimens (based on comparisons between photographs and their specimens; Fig. 10).

The venter of preserved specimens (Fig. 11) varies from grayish tan (e.g., CORBIDI 8234), to dark brown (e.g., CORBIDI 5471, 8305), and black (e.g., CORBIDI 8463, 10018) with white scattered flecks. The throat (Fig. 11) varies from gray (e.g., CORBIDI 8305), grayish tan (e.g., CORBIDI 8229), brown (e.g., CORBIDI 5480, 6694), to dark brown (e.g., CORBIDI 5471, 10018) anteriorly, with white flecks (e.g., CORBIDI 5471, 10019), one white blotch (e.g., CORBIDI 10018) or stripes (e.g., CORBIDI 6694) posteriorly. The subcloacal area is white in most specimens (e.g., CORBIDI 6694, 8229, 8232, 10018), but in some specimens the subcloacal area is black (e.g., CORBIDI 8463).

##### Color in life.

Based on digital photographs (Fig. 10): dorsum varies from tan to brown or reddish brown; creamy tan or mustard brown dorsolaterally; dorsal markings are dark brown, some individuals have brown, creamy, or grayish tan stripes around markings; some individuals also have scattered dark brown flecks dorsolaterally; the flanks are white with black vertical bars; dorsal surfaces of forelimbs and shanks have dark brown transversal bars; the thighs are black with two or three spots on the anterodorsal surfaces. The single suborbital bar is white. The venter is white anteriorly and dark brown or black mottled with translucent gray posteriorly, with white scattered flecks; chest is white and mottled with brown anteriorly; throat is brown or dark brown anteriorly and spotted with white flecks posteriorly in males (posterior part of throat with white blotch with or without stripes in females); the ventral surfaces of the limbs are translucent gray, thighs are mottled with black or dark brown anteriorly and posteriorly the thighs are black with white flecks; the ventral surface of shanks, from the knee to one third or on half the length of the shank, and arms, from the axillae to near the elbow, have a bright amber or orange blotch. Vocal sac in males is olive tan. The iris is creamy white with brown to reddish brown reticulations or reddish brown with creamy white reticulations and a cream ring around pupil.

##### Calls

(Fig. 4C−D). Descriptive statistics of acoustic variables are provided in Table 6. We analyzed recordings from: (1) three males from Tambopata (13.1343°S, 69.6090°W, 233 m) on 5 March 2016, at 19:00h, 21:40h and 24:47h; (2) one male from Reserva Comunal Amarakaeri (12.7834°S, 70.9548°W, 365 m, Madre de Dios Department, Manu Province, Peru) recorded on 5 February 2015; (3) one male from Chontachaka (12.0405°S, 71.7230°W, 630 m, Cusco Department, Paucartambo Province, Peru); and (4) one male from Río Madeira (8.8482°S, 64.0689°W, 110 m, Rondônia State, Brazil) (Lima et al. 2012). Recorded males were not collected.

**Table 6.** Acoustic parameters of *Dendropsophus kamagarini* sp. n. (Southern Clade). Mean ± SD is given with range below. Sample sizes are number of calls. All frequencies are in Hz and durations in s.

	***Dendropsophus kamagarini* sp. n.**					
	**Tambopata (*n* = 4)**	**Amarakaeri (*n* = 1)**	**Chontachaka (*n* = 1)**	**Cobija (*n* = 1)**	**Rio Madeira (*n* = 1)**	**Combined (*n* = 8)**
Advertisement call duration	0.14 ± 0.03 (0.09−0.20)	0.12 ± 0.01 (0.10−0.14)	0.12 ± 0.01 (0.11−0.14)	0.15 ± 0.005 (0.14−0.16)	0.13 ± 0.02 (0.1−0.17)	0.14 ± 0.02 (0.09−0.2)
Advertisement call dominant frequency	3669.6 ± 277.16 (3164.1−4112.8)	3639.1 ± 79.5 (3542.2−3703.7)	4208.19 ± 66.5 (4091.3−4306.6)	3948.1 ± 184.8 (3779.1−4263.6)	3983.6 ± 64.1 (3811.4−4059)	3782.3 ± 286.92 (3164.1−4306.6)
Advertisement call initial frequency	3442.5 ± 246.8 (2964.8−3854.4)	3397.9 ± 59.27 (3316.1−3456.1)	4011.3 ± 153.4 (3886.7−4274.3)	3800.6 ± 51.8 (3671.4−3876)	3746.8 ± 73.5 (3639.1−3854.4)	3562.2 ± 274.4 (2964.8−4274.3)
Advertisement call final frequency	3685.8 ± 277.9 (3175.8−4123.6)	3636.9 ± 69.53 (3542.2−3703.7)	4205.1 ± 69.2 (4080.5−4306.6)	3982.6 ± 175.2 (3779.1−4252.8)	4000.3 ± 45.11 (3929.8−4059)	3798.2 ± 285.91 (3175.8−4306.6)
Advertisement call rise time	0.07 ± 0.01 (0.04−0.1)	0.06 ± 0.01 (0.05−0.07)	0.06 ± 0.004 (0.05−0.07)	0.08 ± 0.002 (0.072−0.079)	0.06 ± 0.01 (0.05−0.08)	0.07 ± 0.01 (0.04−0.1)
Number of pulses of advertisement call	22.8 ± 4.14 (14−32)	16.6 ± 1.52 (15−19)	14.1 ± 1.07 (12−15)	23 ± 1.4 (20−24)	17 ± 2.88 (13−21)	21 ± 4.62 (12−32)
Advertisement call pulse rate	161.91 ± 9.66 (107.69−178.95)	140 ± 5.47 (131.15−145.63)	114.94 ± 7.11 (103.44−125)	151.4 ± 6.4 (140.8−155.8)	132.32 ± 5.23 (123.9−143.9)	151.84 ± 17.18 (103.45−178.94)
Call duration	0.31 ± 0.048 (0.26−0.41)	0.46 ± 0.062 (0.40−0.55)	0.69 ± 0.093 (0.54−0.80)	NA	0.53 ± 0.13 (0.45−0.81)	0.46 ± 0.17 (0.26−0.81)
Inter note interval	0.10 ± 0.011 (0.09−0.13)	0.09 ± 0.014 (0.08−0.11)	0.08 ± 0.009 (0.07−0.09)	NA	0.09 ± 0.006 (0.08−0.01)	0.09 ± 0.01 (0.07−0.13)
Click note duration	0.051 ± 0.009 (0.03−0.067)	0.052 ± 0.017 (0.03−0.082)	0.063 ± 0.02 (0.035−0.10)	NA	0.07 ± 0.008 (0.05−0.08)	0.06 ± 0.01 (0.03−0.10)
Click note dominant frequency	3610.3 ± 267.3 (3164.1−4048.2)	3563.7 ± 62.11 (3445.3−3649.9)	4351.9 ± 75.3 (4242−4532.7)	NA	4024.9 ± 41.86 (3962.1−4102.1)	3981.2 ± 341.9 (3164.1−4532.7)
Number of pulses of click note	6.3 ± 2.2 (1−9)	3.75 ± 2.7 (1−8)	4.5 ± 2.3 (1−10)	NA	7.83 ± 0.91 (6−10)	5.87 ± 2.53 (1−10)
Click note rise time	0.025 ± 0.005 (0.015−0.034)	0.026 ± 0.008 (0.014−0.04)	0.032 ± 0.010 (0.017−0.05)	NA	0.03 ± 0.004 (0.02−0.04)	0.03 ± 0.007 (0.01−0.05)
Click note pulse rate	124.01 ± 34.53 (23.81−151.52)	64.11 ± 33.80 (25.64−112.9)	67.82 ± 19.72 (18.52−111.11)	NA	117 ± 8.11 (91−140.35)	95.6 ± 35.28 (18.52−151.52)
Inter click notes interval	0.08 ± 0.007 (0.07−0.084)	0.085 ± 0.015 (0.069−0.11)	0.068 ± 0.008 (0.054−0.084)	NA	0.07 ± 0.005 (0.06−0.08)	0.07 ± 0.01 (0.05−0.1)

The advertisement call is a pulsed note (Fig. 4C−D). The amplitude of the advertisement call gradually increases until three-quarters of the note duration to decrease abruptly until the end. The advertisement call may be followed or not by secondary click notes. Nonetheless, the click notes are occasionally vocalized alone. The click notes are pulsed except for the last note.

One recording from Cobija, Bolivia (Pando Department, Nicolás Suárez Province) by Márquez et al. (2002) falls within the range of variation of advertisement calls of *Dendropsophus kamagarini* from Peru (Table 6). In addition, the number of pulses (15) and the dominant frequency (4150 Hz) of the call described by Duellman (2005) fall within the range for calls of *D. kamagarini* (Table 6).

##### Distribution and ecology.

*Dendropsophus kamagarini* occurs in the Amazon basin of southeastern Peru (Cusco and Madre de Dios regions; Fig. 9), northwestern Brazil (Acre and Rondônia states; Fig. 9), and northeastern Bolivia, from the Andean slopes to lowland tropical rainforest (Fig. 9). Localities with known elevation range from 150 m (Acre) to 1696 m (Ochigoteni) above sea level.

Bolivian records are partly based on De la Riva et al. (2000) report of “*Dendropsophus parviceps*” in central northeastern Bolivia, Departments of Beni, Cochabamba, La Paz, Pando, and Santa Cruz. One photograph from Puerto Almacén (Santa Cruz Department; pp. 102 in De la Riva et al. 2000) and two photographs from Tahuamanu and El Negro (both from Pando Department; Fig. 10) show the conical tubercle on the upper eyelid characteristic of *D. kamagarini*. *Dendropsophus parviceps* distribution range is at a distance of over 1500 km, which make very unlikely that Bolivian records are conspecific. Thus, we propose that all historic records of “*Dendropsophus parviceps*” from Bolivia are assigned to *D. kamagarini*.

The call from Cobija (Pando Department) falls within the range of advertisement call of *D. kamagarini* (Table 6). The localities of El Negro, Tahuamanu, and Cobija are at a distance of 89 km, 158 km, and 203 km, respectively, to the type locality of *D. kamagarini* (Inotawa). In addition, specimens from Museo de Historia Natural Alcide d’Orbigny, Cochabamba, Bolivia, also have a prominent conical tubercle on the distal edge of the upper eyelid. These specimens are from Valle del Sacta and the confluence of the Altamachi and Ipiri rivers (both from Cochabamba Department; see Appendix 1). There is also one record from Santa Elena (16.6791°S, 66.6791°W, 600 m, Cochabamba Department, Ayopaya Province; Fig. 9) based on a locality record from Museo de Historia Natural Alcide d’Orbigny, Cochabamba, Bolivia. Additionally, the records from Acre, Brazil, of *D. kamagarini* are also supported by Cochran and Goin (1970) who examined one specimen (WCAB 2511) and report the presence of the conical tubercle on the upper eyelid.

*Dendropsophus kamagarini* congregates for breeding at temporary and permanent ponds in flooded forest and Terra Firme forest; it is an opportunistic breeder (Duellman 2005). Adults of both sexes were found at night perching on leaves of bushes and trees, on branches and on palm fronds. Males were calling perched from 2−3 m above the water.

##### Conservation status.

Extent of occurrence (B1) is 637,800 km^2^. *Dendropsophus kamagarini* occurs in the following protected areas from Peru: Otishi National Park, Megantoni National Sanctuary, Amarakaeri Communal Reserve, Manu National Park, Tambopata National Reserve and Bahuaja-Sonene National Park, and protected areas from Bolivia: Manuripi-Heath Amazonian Wildlife National Reserve and Isiboro Sécure National Park and Indigenous Territory. Because its distribution range is large and occurs in several protected areas we suggest that *D. kamagarini* is assigned to the Least Concern category, following IUCN (2001) criteria.

##### Remarks.

The specimens from Cochabamba Department (Appendix 1) are assigned as referred specimens because we lack genetic data. Márquez et al. (1993) report a maximum SVL = 24.6 for males from Puerto Almacén. This value is slightly above the maximum SVL of males of *D. kamagarini* (see Table 3). Márquez et al. (1993) also report the dominant frequency range of the advertisement calls (2476–3144 Hz), which is lower than the dominant frequency range of *D. kamagarini* (3164.1−4306.6 Hz). Therefore, further data is needed to determine the status of that population. We tentatively assign those specimens to *D. kamagarini* as referred material. Schlüter (1979) described the advertisement calls of males from Panguana (Huanáco Department, Peru; Fig. 9), where he reported a dominant frequency range of 3200–4700 Hz and a call duration less than 0.2 s. The frequency range from Panguana is relatively closer to the range of *D. kamagarini* (3164.1−4306.6 Hz) and *D. kubricki* sp. n. (3542.2−4394.5 Hz); however, the call duration (less than 0.2 s) is within the range of calls of *D. kamagarini* (0.09−0.2 s) while the maximum value (0.3 s) of call duration of *D. kubricki* sp. n. exceeds the call duration reported by Schlüter (1979). Thus, we consider the population from Panguana as an unconfirmed register of *D. kamagarini* unless specimen examination demonstrates otherwise. The population from Rio Madeira (Rondônia State, Brazil) is also unconfirmed until specimens are examined.

#### Dendropsophus kubricki sp. n.

Taxon classificationAnimaliaAnuraHylidae

http://zoobank.org/C3677ED1-80E2-412C-A6EE-2949E6C1BB8B

**Figure 13.**
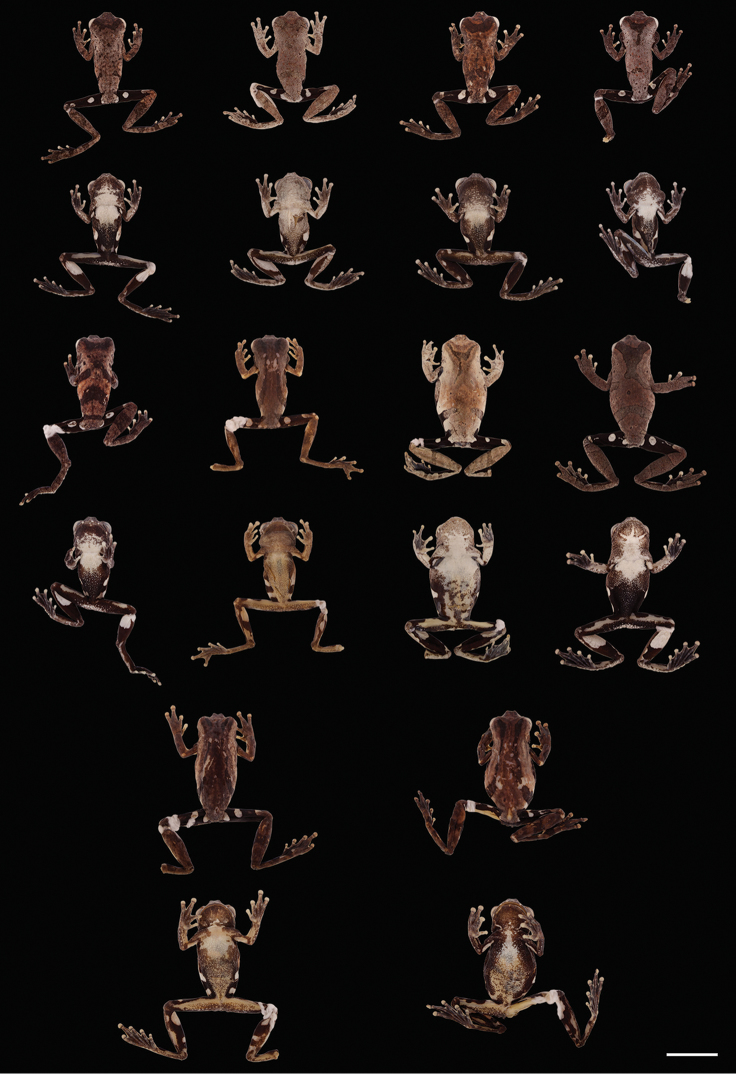
Dorsolateral and ventral views of *Dendropsophus kubricki* sp. n. in life: **A, B** Holotype, adult male, from Río Tapiche, Requena, Peru (CORBIDI 15778) **C, D** Adult male from Río Tapiche, Requena, Peru (CORBIDI 15782) **E** Adult male from Jenaro Herrera, Requena, Peru (not collected) **F** Adults, pair in amplexus from Jenaro Herrera, Requena, Peru (not collected).Dorsolateral and ventral views of *Dendropsophus kubricki* sp. n. in life: **G, H** Adult female from Jenaro Herrera, Requena, Peru (not collected) **I, J** Adult female from Area de Conservación Municipal Chambira, Picota, Peru (CORBIDI 8864) **K** Adult female from Tarapoto, San Martín, Peru (6.4306°S, 76.2903°W, 600 m, not collected) **L** Adults, pair in amplexus from Area de Conservación Municipal Chambira, Picota, Peru (CORBIDI 8864–63). Photographs by P. J. Venegas.

**Figure 14.**
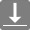
Adults of *Dendropsophus kubricki* sp. n. showing variation in dorsal and ventral coloration of preserved specimens. From left to right, first and second rows: CORBIDI 15780, 15775, 15784, 15777 (males); third and fourth rows: CORBIDI 15779, 8863 (males), 2281, 15785 (females); fifth and sixth rows: CORBIDI 8862, 8864 (females). See Appendix 1 for locality data. Scale bar 10 mm.

Figs 1 , 7C , 13 , 14

##### Holotype.

CORBIDI 15778, an adult male from Peru, Loreto Department, Requena Province, Campamento Wishuincho-Río Tapiche (7.1914°S, 73.9781°W), 120 m above sea level, collected on 11 October 2014 by P. J. Venegas.

##### Paratypes.

Nine adult males and an adult female from Peru, Loreto Department, Requena Province, Campamento Wishuincho-Río Tapiche (7.1914°S, 73.9781°W), 120 m above sea level: CORBIDI 15775–77, adult males, collected on 9 October 2014 by P. J. Venegas; CORBIDI 15779–84, adult males, collected on 12 October 2014 by P. J. Venegas; CORBIDI 15785, adult female, collected on 12 October 2014 by P. J. Venegas. Three adult males and four adult females from Peru, Loreto Department, Requena Province, Sierra del Divisor (6.9187°S, 73.8461°W), 500 m above sea level: CORBIDI 3762, 3970, 3983, adult males, collected in January 2009 by R. Santa Cruz; CORBIDI 2281, adult female, collected on 1 November 2008 by D. Vásquez; CORBIDI 3743–44, 3780, adult females, collected in January 2009 by R. Santa Cruz.

##### Referred specimens.

An adult male and three adult females from Peru, San Martin Department, Picota Province, Área de Conservación Municipal Chambira (7.0375°S, 76.0900°W), 679 m above sea level: CORBIDI 8863, adult male, collected on 27 November 2010 by P. J. Venegas; CORBIDI 8861–62, 8864, adult females, collected on 27 November 2010 by P. J. Venegas.

##### Etymology.

The specific name *kubricki* is a noun in the genitive case and is a patronym for Stanley Kubrick, an American filmmaker who is one of the most brilliant and influential film directors of all time. We dedicate this species to him for his legacy to film culture and science fiction.

##### Diagnosis.

Throughout the species description, coloration refers to preserved specimens unless otherwise noted. The new species is assigned to the genus *Dendropsophus* based on our phylogenetic results (Fig. 1) and the overall similarity with *D. parviceps* and other species of the genus (Figs 13–14). *Dendropsophus kubricki* is a medium-sized species, relative to other species in the *D. parviceps* group and is characterized by the following combination of traits: (1) size sexually dimorphic; mean SVL 19.4 mm in males (range 18.3–20.1; *n* = 14), 26.0 mm in females (range 22.0–28.4; *n* = 8); (2) throat with white flecks posteriorly in males and white blotch with stripes posteriorly in females (Fig. 14); (3) snout truncate in dorsal view, rounded and inclined posteroventrally in lateral view; (4) nostrils slightly prominent; (5) tympanum distinct, rounded, concealed posterodorsally, tympanic membrane non-differentiated and annulus evident; (6) low tubercles on upper eyelid can be distinct or ill-defined; (7) thoracic fold slightly evident or indistinct; (8) ulnar tubercles and outer tarsal tubercles low; (9) axillary membrane present; (10) skin on dorsal surfaces smooth with scattered tubercles mainly on head; skin on throat areolate, skin on chest, belly, posterior surfaces of thighs, and subcloacal area coarsely areolate; (11) dark brown markings on dorsum consisting of chevrons and transverse blotches in variable arrangements (Fig. 14); (12) thenar tubercle distinct; (13) hand webbing formula II1-−2^+^III1-−1-IV, foot webbing formula I1-−2-II1-−2-III1-–2IV2−1-V; (14) in life, dorsal surfaces reddish brown, brown, or grayish tan; (15) orange to amber blotch on the proximal ventral surface of shanks and under arms, from the axillae to near elbow, in life (white to creamy white in preserved); (16) one suborbital white bar present both in life and preserved; (17) anterodorsal surfaces of thighs are black to dark brown with two or three white spots, both in life and preserved; (18) iris in life is reddish brown, brown or silver gray.

##### Comparisons with other species.

*Dendropsophus kubricki* is most similar to *D. kamagarini* and *D. parviceps*. It is distinguished from *D. parviceps* by its larger size (Fig. 2; see Morphological comparisons), lower dominant frequency in advertisement call (Fig. 4A–B, E–F; see Bioacoustic comparisons), throat with white flecks or white medial spot posteriorly in males, both in life and preservative (dark flecks posteriorly in males both in life and preservative in *D. parviceps*), and scattered low tubercles on the upper eyelids (smooth in *D. parviceps*). *Dendropsophus kubricki* differs from *D. kamagarini* by lacking a prominent conical tubercle on the distal edge of the upper eyelid (present in *D. kamagarini*; Fig. 12). *Dendropsophus kubricki* also differs from *D. parviceps* and *D. kamagarini* by having a more rounded snout in profile (Fig. 12). *Dendropsophus kubricki* differs from other species of the *D. parviceps* group (*sensu* Fouquet et al. 2015) by having, in life, an orange blotch on the proximal ventral surface of shanks and arms [orange blotches are absent in *D. bokermanni* (Goin 1960; Duellman and Crump 1974), in *D. brevifrons* (Duellman and Crump 1974), in *D. counani* (Fouquet et al. 2015), in *D. frosti* (Motta et al. 2012) and in *D. koechlini* (Duellman and Trueb 1989)]. *Dendropsophus kubricki* also resembles *D. pauiniensis*, but it differs by the presence of an orange blotch on the proximal ventral surface of shanks (absent in *D. pauiniensis*; Heyer 1977).

##### Description of holotype.

Adult male (Fig. 7C), SVL 19.0 mm. Head as wide as body, slightly wider than long, widest below eyes; snout truncate and short in dorsal view, moderately rounded and slightly inclined posteroventrally in lateral view; loreal region concave; lips thin; internarial region slightly concave; nostrils slightly protuberant dorsally and laterally; interorbital area flat; tympanum rounded and distinct, tympanic annulus evident, tympanic membrane non-differentiated, supratympanic fold thin, covering tympanum posterodorsally. Arms slender, not hypertrophied; axillary membrane extending along proximal one third of arm; ulnar fold distinct, low ulnar tubercles present; fingers short, bearing small, round discs; relative length of fingers I < II < IV < III; subarticular tubercles small, round on fingers I and II, bifid on finger III, and divided on finger IV; supernumerary tubercles small, slightly evident; thenar tubercle distinct; palmar tubercle flat, round; webbing basal between fingers I and II; hand webbing formula II1-−2-III1-–1IV. Hindlimbs long, slender; tarsal fold absent, low outer tarsal tubercles present; calcar and heel tubercles absent; toes moderately long, bearing round discs, smaller than those of fingers; relative length of toes I < II < III < V < IV; subarticular tubercles small, round; supernumerary tubercles indistinct; inner metatarsal tubercle small, flat, elliptical; outer metatarsal tubercle absent; foot webbing formula I1-–2II1-–2III1-–2IV2–1-V. Skin on dorsum and head smooth with scattered tubercles, skin on dorsal surfaces of limbs, flanks and groins smooth; skin on venter, posterior surfaces of thighs and subcloacal area coarsely areolate; skin on chest and throat areolate; skin on other surfaces smooth. Cloacal opening directed posteriorly at upper level of thighs; cloacal sheath short; cloacal folds and tubercles absent. Tongue cordiform, barely free posteriorly; dentigerous process of vomers small, prominent, adjacent medially, each bearing three and five vomerine teeth (left/right), positioned obliquely to choanae; choanae small, rounded; vocal slits long, extending from midlateral base of tongue to angle of jaws; vocal sac single, median, subgular.

##### Color of holotype in preservative

(Fig. 7C). Dorsal surfaces of head, body, and limbs brownish gray with scattered reddish brown flecks with melanophores and leucophores on dorsum, dark brown markings on dorsum consisting of a median blotch anteriorly and transverse bar posteriorly; dark brown broad transverse bars on the forelimbs and shanks; snout brown dorsally; scapular region gray; thighs black with three white spots on the anterodorsal surfaces; one small white suborbital bar. Ventral surface of belly dark brown mottled with white anteriorly and with translucent white posteriorly and laterally; chest white; throat dark brown anteriorly and white with dark brown flecks posteriorly; ventral surfaces of the forelimbs translucent white; thighs translucent white anteriorly and dark brown posteriorly.

##### Measurements of holotype (in mm).

SVL 19.0, HW 6.6, HL 6.3, END 1.8, IN 1.5, FL 9.1, TL 10.0, FL 7.3.

##### Variation.

Morphometric variation of the paratype series is summarized in Table 3. Variation in dorsal and ventral coloration of preserved specimens is shown in Figure 14. Dorsal coloration in alcohol varies from gray (e.g., CORBIDI 15777) to grayish tan (e.g., CORBIDI 2281, 15775), reddish brown (e.g., CORDIBI 15779), brown (e.g., CORBIDI 8864), grayish brown (e.g., CORBIDI 15780), dark brown (e.g., CORBIDI 8862), or pinkish gray (e.g., CORBIDI 15785); some specimens have scattered reddish brown low tubercles and slightly black flecks (e.g., CORBIDI 15775, 15777, 15784). Occipital region with dark brown median blotch, one blotch in scapular region and dark brown transverse bar in sacral region extending onto flanks (e.g., CORBIDI 15779, 15780, 15784); some specimens have two “)(” shaped dorsolateral stripes beginning on the upper eyelids and ending on the posterior flanks (e.g., CORBIDI 8862, 8864). The dorsum has tubercles mainly on head and upper eyelid (e.g., CORBIDI 8864, 8861, 15776, 15785), but some specimens have a smooth dorsum without tubercles (e.g., CORBIDI 2281, 3744; Fig. 14). Some specimens have postrictal tubercles, posteroventrally to tympanic annulus (e.g., CORBIDI 15775, 15780).

The venter of preserved specimens (Fig. 14) varies from black (e.g., CORBIDI 15780, 15785) to dark brown (e.g., CORBIDI 8864) with scattered white or creamy flecks. The throat anteriorly (Fig. 14) varies from brown (e.g., CORBIDI 8863, 15784), dark brown (e.g., CORBIDI 8862), grayish tan (e.g., CORBIDI 2281, 15775) to black (e.g., CORBIDI 15779, 15780) with white flecks (e.g., CORBIDI 15779), one irregular white stain (e.g., CORBIDI 2281, 8862) or stripes (e.g., CORBIDI 8864, 15785) posteriorly. The subcloacal area is white in most specimens (e.g., CORBIDI 15775, 15780, 15785), but it is black in some (e.g., CORBIDI 8862, 8863, 8864).

##### Color in life.

Based on digital photographs (Fig. 13): dorsum varies from reddish brown, brownish gray, brown to creamy tan suffused with gray and brown with scattered white flecks, with or without reddish brown or brown low tubercles; some individuals have few scattered dark brown flecks on the dorsum; flanks are white with black or dark brown vertical bars; dorsal markings are dark brown, some individuals are mustard brown dorsolaterally; dorsal surfaces of forelimbs and shanks have dark brown transversal bars; anterodorsal surfaces of the thighs are black to dark brown with two or three spots. The single suborbital bar is white. The venter is black to dark brown mottled with translucent gray, and with white flecks anteriorly; some individuals have scattered white flecks on venter; chest is white; throat is brown, reddish brown, or dark brown with white flecks posteriorly in males (posterior part of throat with white blotch with or without stripes in females), some individuals have a white medial spot adjacent to the chest; the ventral surfaces of the limbs are translucent gray, the thighs are mottled with black or dark brown anteriorly; posteriorly the thighs are black with white flecks; the proximal ventral surface of shanks and arms, from the axillae to near the elbow, have an orange or amber blotch. The iris is reddish brown, brown, or silver gray.

##### Calls

(Fig. 4E−F). Descriptive statistics of acoustic variables are provided in Table 7. We recorded three males (two not collected and CORBIDI 15778) at Campamento Wishuincho-Río Tapiche (Loreto Department, Requena Province, Peru) on 11 October 2014 at 12:53h and 12 October 2014 at 21:53h. We also recorded one male (not collected) at Cordillera Azul (7.8103°S, 75.9928°W, 725 m, San Martín Department, Picota Province, Peru).

**Table 7.** Acoustic parameters of *Dendropsophus kubricki* sp. n. (Central Clade). Mean ± SD is given with range below. Sample sizes are number of calls. All frequencies are in Hz and durations in s.

	***Dendropsophus kubricki* sp. n.**		
	**Río Tapiche (*n* = 3)**	**Cordillera Azul (*n* = 1)**	**Combined (*n* = 4)**
Advertisement call duration	0.13 ± 0.02 (0.1−0.16)	0.23 ± 0.04 (0.13−0.3)	0.19 ± 0.06 (0.1−0.3)
Advertisement call dominant frequency	4062.6 ± 248.78 (3691.4−4394.5)	3998.9 ± 137.88 (3542.2−4242)	4024.7 ± 191.88 (3542.2−4394.5)
Advertisement call initial frequency	3722 ± 261.11 (3222.7−4066.4)	3664.9 ± 182 (3380.7−4015.9)	3688.1 ± 217.86 (3222.7−4066.4)
Advertisement call final frequency	4066.7 ± 250.85 (3691.4−4394.5)	4026.7 ± 105.17 (3703.7−4242)	4042.9 ± 178.72 (3691.4−4394.5)
Advertisement call rise time	0.06 ± 0.008 (0.05−0.08)	0.12 ± 0.02 (0.06−0.15)	0.09 ± 0.03 (0.05−0.15)
Number of pulses of advertisement call	17.76 ± 2.47 (14−22)	27 ± 6.16 (14−27)	23.26 ± 6.24 (14−34)
Advertisement call pulse rate	140.76 ± 5.27 (133.33−158.27)	129.05 ± 34.18 (110.6−228.57)	133.79 ± 27.1 (110.6−228.57)
Call duration	0.42 ± 0.11 (0.23−0.63)	0.54 ± 0.06 (0.45−0.59)	0.44 ± 0.12 (0.23−0.63)
Inter note interval	0.076 ± 0.013 (0.05−0.10)	0.08 ± 0.006 (0.075−0.088)	0.08 ± 0.011 (0.05−0.10)
Click note duration	0.051 ± 0.011 (0.03−0.07)	0.073 ± 0.009 (0.051−0.09)	0.06 ± 0.014 (0.03−0.09)
Click note dominant frequency	4069.2 ± 269.6 (3703.1−4500)	4023.3 ± 32.82 (3962.1−4080.5)	4057.4 ± 233.03 (3703.1−4500)
Number of pulses of click note	4.5 ± 1.71 (1−7)	6.4 ± 1.3 (2−7)	5 ± 1.8 (1−7)
Click note rise time	0.026 ± 0.005 (0.014−0.03)	0.04 ± 0.005 (0.026−0.04)	0.028 ± 0.007 (0.014−0.04)
Click note pulse rate	85.84 ± 21.27 (27.03−117.6)	87.66 ± 15.0 (39.22−111.11)	86.3 ± 19.7 (27.03−117.6)
Inter click notes interval	0.083 ± 0.012 (0.058−0.10)	0.080 ± 0.0092 (0.066−0.095)	0.082 ± 0.011 (0.06−0.10)

**Table 8.** Character loadings and eigenvalues for Principal Components (PC) I–II. The analysis was based on seven acoustic variables from advertisement calls of *Dendropsophus parviceps* (Northern Clade), *D. kamagarini* sp. n. (Southern Clade), and *D. kubricki* sp. n. (Central Clade). Bold numbers indicate highest loadings.

**Variables**	**PCA Advertisement call**	
	**PCI**	**PCII**
Note duration	-0.24	**0.58**
Dominant frequency	**0.48**	0.17
Initial frequency	**0.48**	0.16
Final frequency	**0.48**	0.17
Rise time	-0.21	**0.59**
Number of pulses	-**0.40**	0.20
Pulse rate	-0.23	-**0.43**
Eingevalue	3.80	2.34
% of variation	54.2	33.5

The advertisement call is a pulsed note (Fig. 4E−F). The amplitude of the advertisement call increases gradually until three-quarters of the note duration to decrease abruptly until the end. This call may be followed by one or more click notes. These clicks sometimes are emitted alone. Moreover, the click notes are pulsed, except for the last one which is unpulsed.

##### Distribution and ecology.

*Dendropsophus kubricki* is distributed in the Amazon basin in northeastern and central Peru (Fig. 9), at elevations between 106 (Jenaro Herrera) and 725 m (Cordillera Azul). *Dendropsophus kubricki* was found in flooded forest. Specimens from Chambira were collected in a small pond in a Terra Firme forest. Males call at night while perching on leaves of bushes and trees. They were observed between 0.3 and 0.4 m above the water.

##### Conservation status.

Extent of occurrence (B1) is 53,548 km^2^. *Dendropsophus kubricki* occurs in the following protected areas: Sierra del Divisor National Park, Cordillera Azul National Park, and Cordillera Escalera Regional Conservation Area. Because its distribution range is large and occurs in protected areas, we recommend that *D. kubricki* is assigned to the Least Concern category, following IUCN (2001) criteria.

##### Remarks.

Specimens from Chambira (Picota Province) are closely related to Río Tapiche and Jenaro Herrera specimens (both localities from Requena Province) (Fig. 1); genetic distances between these populations are low (mean p-distances 1.3% in mitochondrial gene 12S). Therefore, we include them tentatively in *Dendropsophus kubricki*. However, individuals from Chambira and Tarapoto (Picota and San Martin provinces, respectively) show differences in coloration because their dorsal tubercles have the same color as the background, white flecks and reddish brown low tubercles on the dorsum and dorsal surfaces are absent both in life and preservative (present in Río Tapiche and Jenaro Herrera individuals; Figs 13–14). In addition, there is segregation in acoustic space between advertisement calls from Cordillera Azul (Picota Province) and the type locality (Fig. 5; Table 7). Therefore, it is conceivable that they represent a separate species.

## Discussion

Our genetic, morphologic, and bioacoustic data demonstrated that *Dendropsophus parviceps*, as previously defined, was a complex of three cryptic species. Duellman (2005) noted the morphological distinctiveness of populations from Peru and suggested that they may represent separate species. However, without genetic information the definition of species limits within “*D. parviceps*” was difficult because the morphology of the three species is highly conserved. The dark brown markings on dorsum, the suborbital bar, spots on the anterior surfaces of thighs, and orange or amber blotches on shanks and arms are shared between the three species. Differences between *D. parviceps*, *D. kamagarini*, and *D. kubricki* are limited to body size, skin texture, and advertisement calls. This pattern of highly conserved morphology has also been reported in other species complexes in Amazonian amphibians (e.g., Caminer and Ron 2014; Elmer et al. 2007; Fouquet et al. 2012; Funk et al. 2012; Kieswetter and Schneider 2013; Moravec et al. 2014; Rojas et al. 2016).

The pattern of variation in bioacoustic, and quantitative and qualitative morphological characters found in the *D. parviceps* species complex is not unusual among closely related species of Amazonian amphibians. Genetic divergence usually covaries with size, bioacoustic, and qualitative morphological characters (e.g., skin ornamentation and coloration) while it has low covariation with size-corrected morphometric variables (Caminer et al. 2017; dos Santos et al. 2015; Fouquet et al. 2012; Funk et al. 2012; Ortega-Andrade et al. 2015; Ron et al. 2016, but see Acevedo et al. 2016). This suggests that advertisement calls and qualitative morphological characters are among the first components of the phenotype to diverge during speciation. In contrast, body shape, as quantified in linear morphometric analyses, is highly conserved and of limited value to assess limits among closely related species.

### Speciation modes

Several authors have discussed the role of niche evolution in the speciation of vertebrates in tropical mountains (e.g., Cadena et al. 2012; Kozak and Wiens 2007). Some studies have shown that sister species tend to segregate along environmental gradients suggesting ecological speciation and niche lability (Arteaga et al. 2016; Graham et al. 2004; Ron et al. 2012). Other studies have shown that sister species tend to be allopatric but with similar environmental niches (Cadena et al. 2012; Ortega-Andrade et al. 2015). Those results imply vicariant speciation and niche conservativism. Clearly, both speciation mechanisms have contributed to the high diversity of the Andes and adjacent Amazon lowlands. Our results with the *Dendropsophus parviceps* complex suggest niche conservativism and vicariance speciation. We base this conjecture in the elevational distribution of the three species and in the intraspecfic genetic variation among populations of *D. parviceps*.

*Dendropsophus parviceps* and *D. kamagarini* show wide and overlapping elevation ranges: 151 m to 1600 m in *D. parviceps* and 150 m to 1696 m in *D. kamagarini*. Fewer localities are known for *D. kubricki* but its known range (106–725 m) overlaps with the ranges of the other two species. Because elevation is the most influential variable defining the environmental niche in tropical regions, overlapping elevation ranges suggest conserved environmental niches. The allopatric distribution of the three species also indicates that vicariant speciation with latitudinal replacement is more likely than ecological speciation with elevational replacement.

The lack of importance of elevation in promoting genetic differentiation is also suggested by interpopulation genetic differentiation in *D. parviceps*. We sampled 55 populations encompassing an elevation range of 186–1600 m. If disruptive selection across the elevation gradient were generating genetic isolation, we would expect to find parapatric clades segregating by elevation. Instead, two parapatric clades were found that segregate latitudinally, each occurring across a wide range of elevations. Both clades have a contact zone in central Amazonia, Ecuador (Fig. 1). A northern subclade occurs in Napo, Orellana, Sucumbíos provinces reaching marginally Pastaza Province; a southern subclade occurs in Pastaza, Tungurahua, and Amazonian Peru. Both clades have a narrow zone of contact near the limit between Napo and Pastaza provinces at an elevation of 900 m. Genetic distances between both clades are moderate (range for gene 12S is 0.4–1.2%), but there is strong support for each clade indicating structured genetic differentiation. Currently, there are not conspicuous geographic barriers between both clades leaving as an open question the processes that promoted genetic divergence within *D. parviceps*. Overall, the available evidence suggests that species of the *D. parviceps* complex speciated allopatrically instead of ecologically along an elevation gradient.

## Supplementary Material

XML Treatment for Dendropsophus parviceps

XML Treatment for Dendropsophus kamagarini

XML Treatment for Dendropsophus kubricki

## Appendix 1

**Specimens examined**

*Dendropsophus parviceps*

ECUADOR: PROVINCIA SUCUMBIOS: Puerto Bolívar (0.0886°S, 76.1420°W), 240 m (QCAZ 28247, 28249); Playas Cuyabeno (0.2415°S, 75.9305°W), 230 m (QCAZ 28269, 28399); Rey de los Andes (0.2082°S, 76.2369°W), 270 m (QCAZ 28455, 28492); Reserva Limoncocha (0.4062°S, 76.6194°W), 261 m (QCAZ 43140, 44915); La Selva Lodge (0.4982°S, 76.3738°W), 229 m (QCAZ 4340, 44055, 47736); Río Napo, Pañacocha (0.4712°S, 76.0667°W), 225 m (QCAZ 44140); Zábalo (0.3181°S, 75.7662°W), 220 m (QCAZ 27983); Garza Cocha (0.4816°S, 75.3442°W), 195 m (CORBIDI 15). PROVINCIA NAPO: Río Hollín (0.6958°S, 77.7303°W), 1068 m (QCAZ 17942, 22208); Sumaco (0.6866°S, 77.6013°W), 1430 m (QCAZ 48919, 48929, 48931); Estación Biológica Jatun Sacha (1.0650°S, 77.6142°W), 397 m (QCAZ 18177, 18184); Ahuano (1.0546°S, 77.5484°W), 385 m (QCAZ 27028). PROVINCIA ORELLANA: Río Napo, Chiroisla, Banco sur (0.5799°S, 75.9177°W), 203 m (QCAZ 44355−57); Río Napo, Santa Teresita (0.9009°S, 75.4136°W), 186 m (QCAZ 44734−36); Río Napo, Huiririma, (0.7116°S, 75.6239°W), 194 m (QCAZ 44599−601); Río Napo, Nuevo Rocafuerte, (0.9193°S, 75.4010°W), 187 m (QCAZ 44775, 44778); Río Napo, Chiroisla, (0.5756°S, 75.8998°W), 203 m (QCAZ 44440−42); Río Napo, Eden, Banco sur (0.4983°S, 76.0711°W), 216 m (QCAZ 44227−29); Río Napo, San Vicente (0.6790°S, 75.6511°W), 196 m (QCAZ 44492−94); Río Napo, Áñangu (0.5249°S, 76.3844°W), 255 m (QCAZ 43968−69); Coca, Río Napo (0.4778°S, 76.9898°W), 267 m (QCAZ 43680, 43704); Coca, Banco sur Río Napo (0.4989°S, 77.0075°W), 264 m (QCAZ 43775); Río Napo, Primavera (0.4310°S, 76.7865°W), 244 m (QCAZ 43892); Parque Nacional Yasuní, Estación Científica Yasuní, Pontificia Universidad Católica del Ecuador (0.6744°S, 76.3970°W), 250 m (QCAZ 35720, 51075, 51229−30); Parque Nacional Yasuní, Pompeya-Iro road, 80−75 km (0.8401°S, 76.3024°W), 250 m (QCAZ 43066−67); Parque Nacional Yasuní. Pompeya-Iro, 96 km (0.9065°S, 76.2214°W), 233 m (QCAZ 51195); Parque Nacional Yasuní, Pozo SPF, 8 km (0.6916°S, 75.9196°W), 250 m (QCAZ 31267). PROVINCIA PASTAZA: Bobonaza (1.4981°S, 77.8793°W), 660 m (QCAZ 32491); Cononaco (1.2083°S, 76.7167°W), 220 m (QCAZ 38948); Fátima (1.4114°S, 78.0000°W), 1023 m (QCAZ 15424, 49334); Bobonaza, Tuculí (1.4945°S, 77.8696°W), 620 m (QCAZ 32543); Zanjarajuno (1.3572°S, 77.8705°W), 977 m (QCAZ 49314, 49340); Arajuno (1.3243°S, 77.6890°W), 580 m (QCAZ 38332); Canelos (1.6065°S, 77.7576°W), 465 m (QCAZ 52816, 52820); Canelos-Puyo road (1.6016°S, 77.7576°W), 465 (QCAZ 52837−38); Sarayaku, Río Palandayaku (1.7355°S, 77.4902°W), 325 m (QCAZ 52751−53, 52755); Montalvo, Comunidad Campus (1.9924°S, 76.9168°W), 392 m (QCAZ 53161, 53165−67, 53181−82, 53184); Killu Allpa (2.1871°S, 76.8577°W), 335 m (QCAZ 52892); Parque Nacional Llanganates, Comunidad Zarentza (1.3587°S, 78.0511°W), 1338 m (QCAZ 59772). PROVINCIA TUNGURAHUA: Río Verde (1.4001°S, 78.3006°W), 1600 m (QCAZ 52017−19); Río Negro (1.4125°S, 78.2042°W), 1244 m (QCAZ 52023−24, 52026). PERU: REGION LORETO: PROVINCIA DATEM DEL MARAÑÓN: Andoas (2.6516°S, 76.5137°W), 151 m (CORBIDI 1039−41, 1044, 1046, 1059, 5029). PROVINCIA LORETO: San Jacinto (2.3308°S, 75.8637°W), 160 m (CORBIDI 1144, 1151−52).

***Dendropsophus kamagarini***

PERU: DEPARTAMENTO MADRE DE DIOS: PROVINCIA TAMBOPATA: Inotawa (12.8092°S, 69.3182°W), 192 m (CORBIDI 5246); La Habana (12.6537°S, 69.1796°W), 192 m (CORBIDI 5259). DEPARTAMENTO CUSCO: PROVINCIA LA CONVENCIÓN: Comunidad Nativa Poyentimari (12.1885°S, 73.0009°W), 725 m (CORBIDI 8150–53, 8228–36, 8285–86, 8305, 8463, 8476); Megantoni (12.2581°S, 72.8425°W), 670 m (CORBIDI 6659, 6664, 6679, 6685, 6687–88, 6692, 6694, 6698); Comunidad Nativa Chokoriari (11.9569°S, 72.9409°W), 434 m (CORBIDI 8067–70); Puyantimari (12.1861°S, 73.0004°W), 710 m (CORBIDI 9762); Pongo de Mainique (12.2581°S, 72.8425°W), 670 m (CORBIDI 5471, 5473, 5480, 5484); Pagoreni norte (11.7115°S, 73.8967°W), 402 m (CORBIDI 10018–19); Comunidad Ochigoteni (12.5758°S, 73.09°W), 1696 m (CORBIDI 5392); Palmeiras-Alto Shimá (12.5453°S, 73.1350°W), 500 m (CORBIDI 10585); Chokoriari (11.9569°S, 72.9409°W), 413 m (CORBIDI 10628). BOLIVIA: DEPARTAMENTO COCHABAMBA: PROVINCIA AYOPAYA: Confluence of the Altamachi and Ipiri rivers (16.0543°S, 66.6667°W), 600 m (MHNC-A 427, 429). PROVINCIA CARRASCO: Valle del Sacta (17.118°S, 64.767°W), 230 m (MHNC-A 2116).

***Dendropsophus kubricki***

PERU: DEPARTAMENTO LORETO: PROVINCIA REQUENA: Sierra del Divisor (6.9187°S, 73.8461°W), 500 m (CORBIDI 2281, 3743–44, 3762, 3780, 3970, 3983); Campamento Wishuincho-Río Tapiche (7.1914°S, 73.9781°W), 120 m (CORBIDI 15775–85). DEPARTAMENTO SAN MARTÍN: PROVINCIA PICOTA: Área de Conservación Municipal Chambira (7.0375°S, 76.0900°W), 679 m (CORBIDI 8861–64).

## Appendix 2

**Table:** GenBank accession numbers for DNA sequences used in the phylogenetic analyses. Abbreviations: BRA = Brazil, CO = Colombia, EC = Ecuador, PE = Peru.

Museum number	Species	Locality	Alt. (m)	Latitude	Longitude	Genbank Accession numbers		
						12S	16S-ND1-tRNA	CO1
QCAZ 28247	*D. parviceps*	Sucumbíos, Puerto Bolívar. EC	240	-0.0886, -76.1420	MG041769	MG041894	MG041832
QCAZ 28492	*D. parviceps*	Sucumbíos, Rey de los Andes. EC	270	-0.2082, -76.2369	MG041770	MG041895	MG041831
QCAZ 28455	*D. parviceps*	Sucumbíos, Rey de los Andes. EC	270	-0.2082, -76.2369	MG041771	MG041896	MG041830
QCAZ 28399	*D. parviceps*	Sucumbíos, Cuyabeno. EC	230	-0.2415, -75.9305	MG041768	MG041892	MG041833
QCAZ 28269	*D. parviceps*	Sucumbíos, Cuyabeno. EC	230	-0.2415, -75.9305	MG041767	MG041893	MG041834
QCAZ 27983	*D. parviceps*	Sucumbíos, Zábalo. EC	220	-0.3181, -75.7662	MG041772	MG041897	MG041835
QCAZ 28249	*D. parviceps*	Sucumbíos, Puerto Bolívar. EC	240	-0.0886, -76.1420	MG041773	MG041898	MG041836
QCAZ 44915	*D. parviceps*	Sucumbíos, Limoncocha. EC	261	-0.4062, -76.6195	MG041774	MG041899	MG041837
QCAZ 47736	*D. parviceps*	Sucumbíos, La Selva. EC	229	-0.4982, -76.3738	MG041775	MG041900	MG041838
QCAZ 31267	*D. parviceps*	Orellana, PNY, Pozo SPF, 8 km. EC	250	-0.6916, -75.9196	MG041776	MG041901	MG041839
QCAZ 43066	*D. parviceps*	Orellana, PNY, Pompeya-Iro road, 80−75 km. EC	250	-0.8401, -76.3024	MG041777	MG041902	MG041840
QCAZ 43067	*D. parviceps*	Orellana, PNY, Pompeya-Iro road, 80−75 km. EC	250	-0.8401, -76.3024	MG041778	MG041903	MG041841
QCAZ 43680	*D. parviceps*	Orellana, Coca, northern Río Napo. EC	267	-0.4778, -76.9898	MG041779	MG041904	MG041842
QCAZ 43892	*D. parviceps*	Orellana, Primavera. EC	244	-0.431, -76.7865	MG041780	MG041905	MG041843
QCAZ 43969	*D. parviceps*	Orellana, Áñangu. EC	255	-0.5249, -76.3844	MG041781	MG041906	MG041844
QCAZ 44734	*D. parviceps*	Orellana, Santa Teresita. EC	186	-0.9009, -75.4136	MG041782	MG041907	MG041845
QCAZ 43775	*D. parviceps*	Orellana, Coca, southern Río Napo. EC	264	-0.4989, -77.0075	MG041783	MG041908	MG041846
QCAZ 51229	*D. parviceps*	Orellana, PNY, Estación Científica Yasuní, PUCE. EC	250	-0.6744, -76.3970	MG041784	MG041909	MG041847
QCAZ 44355	*D. parviceps*	Orellana, Chiroisla, southern Río Napo. EC	207	-0.5799, -75.9177	MG041785	MG041910	MG041848
QCAZ 44492	*D. parviceps*	Orellana, San Vicente. EC	196	-0.6790, -75.6511	MG041786	MG041911	MG041849
QCAZ 44600	*D. parviceps*	Orellana, Huiririma. EC	194	-0.7116, -75.6239	MG041787	MG041912	MG041850
QCAZ 44735	*D. parviceps*	Orellana, Santa Teresita. EC	186	-0.9009, -75.4136	MG041788	MG041913	MG041851
QCAZ 44228	*D. parviceps*	Orellana, Edén. EC	216	-0.4983, -76.0711	MG041789	MG041914	MG041852
QCAZ 44227	*D. parviceps*	Orellana, Edén. EC	216	-0.4983, -76.0711	MG041790	MG041915	MG041853
QCAZ 44442	*D. parviceps*	Orellana, Chiroisla, northern Río Napo. EC	203	-0.5756, -75.8998	MG041791	MG041916	MG041854
QCAZ 51230	*D. parviceps*	Orellana, PNY, Estación Científica Yasuní, PUCE. EC	250	-0.6744, -76.3970	MG041792	MG041917	MG041855
QCAZ 51195	*D. parviceps*	Orellana, PNY, Pompeya-Iro, 96 km. EC	233	-0.9065, -76.2214	MG041793	MG041918	MG041856
QCAZ 44778	*D. parviceps*	Orellana, Nuevo Rocafuerte. EC	187	-0.9193, -75.4010	MG041794	MG041919	MG041857
QCAZ 18184	*D. parviceps*	Napo, Estación Biológica Jatun Sacha. EC	397	-1.0650, -77.6142	MG041795	NA	NA
QCAZ 48931	*D. parviceps*	Napo, Sumaco. EC	1430	-0.6866, -77.6013	MG041796	MG041920	MG041858
QCAZ 18177	*D. parviceps*	Napo, Estación Biológica Jatun Sacha. EC	397	-1.0650, -77.6142	MG041797	MG041921	MG041859
QCAZ 22208	*D. parviceps*	Napo, Río Hollín. EC	1068	-0.6958, -77.7303	MG041798	MG041922	MG041860
QCAZ 17942	*D. parviceps*	Napo, Río Hollín. EC	1068	-0.6958, -77.7303	MG041799	MG041923	MG041861
QCAZ 48919	*D. parviceps*	Napo, Sumaco. EC	1430	-0.68659, -77.60133	MG041800	MG041924	MG041862
QCAZ 49340	*D. parviceps*	Pastaza, Zanjarajuno. EC	977	-1.3572, -77.8706	MG041801	MG041925	MG041863
QCAZ 52024	*D. parviceps*	Tungurahua, Río Negro. EC	1244	-1.4125, -78.2042	MG041802	NA	MG041864
QCAZ 52026	*D. parviceps*	Tungurahua, Río Negro. EC	1244	-1.4125, -78.2042	MG041803	MG041926	MG041865
QCAZ 52017	*D. parviceps*	Tungurahua, Río Verde. EC	1600	-1.4001, -78.3006	MG041804	MG041927	MG041866
QCAZ 52018	*D. parviceps*	Tungurahua, Río Verde. EC	1600	-1.4001, -78.3006	MG041805	MG041928	MG041867
QCAZ 52753	*D. parviceps*	Pastaza, Sarayaku, Río Palandayaku. EC	325	-1.7355, -77.4902	MG041806	NA	MG041868
QCAZ 32543	*D. parviceps*	Pastaza, Tuculí. EC	620	-1.4945, -77.8696	MG041807	MG041929	MG041869
QCAZ 32555	*D. parviceps*	Pastaza, Tuculí. EC	620	-1.4945, -77.8696	MG041808	MG041930	MG041870
QCAZ 52755	*D. parviceps*	Pastaza, Sarayaku, Río Palandayaku. EC	325	-1.7355, -77.4902	MG041809	MG041931	MG041871
QCAZ 49334	*D. parviceps*	Pastaza, Fátima. EC	1023	-1.4114	-78	MG041810	MG041932	MG041872
QCAZ 52838	*D. parviceps*	Pastaza, Canelos-Puyo road. EC	465	-1.6016, -77.7576	MG041811	MG041933	MG041873
QCAZ 52816	*D. parviceps*	Pastaza, Canelos. EC	465	-1.6065, -77.7536	MG041812	MG041934	MG041874
QCAZ 52752	*D. parviceps*	Pastaza, Sarayaku, Río Palandayaku. EC	325	-1.7355, -77.4902	MG041813	MG041935	MG041875
QCAZ 52751	*D. parviceps*	Pastaza, Sarayaku, Río Palandayaku. EC	325	-1.7355, -77.4902	MG041814	MG041936	MG041876
QCAZ 53161	*D. parviceps*	Pastaza, Montalvo. EC	392	-1.9924, -76.9168	MG041815	MG041937	MG041877
QCAZ 52892	*D. parviceps*	Pastaza, Killu Allpa. EC	335	-2.1871, -76.8577	MG041816	MG041938	MG041878
QCAZ 32491	*D. parviceps*	Pastaza, Bobonaza. EC	660	-1.4981, -77.8793	MG041817	MG041939	MG041879
QCAZ 53167	*D. parviceps*	Pastaza, Montalvo. EC	392	-1.9924, -76.9168	MG041818	MG041940	MG041880
QCAZ 53183	*D. parviceps*	Pastaza, Montalvo. EC	392	-1.9924, -76.9168	MG041819	MG041941	MG041881
QCAZ 38332	*D. parviceps*	Pastaza, Arajuno. EC	580	-1.3243, -77.689	MG041820	MG041942	MG041882
QCAZ 39515	*D. parviceps*	Pastaza, Cononaco. EC	220	-1.2083, -76.7167	MG041821	MG041943	MG041883
CORBIDI 8150	*D. kamagarini*	La Convencion, Poyentimari. PE	725	-12.1885, -73.0009	MG041822	NA	MG041884
CORBIDI 5471	*D. kamagarini*	La Convencion. Pongo de Mainique. PE	670	-12.2581, -72.8425	MG041823	MG041944	MG041885
CORBIDI 5246	*D. kamagarini*	Tambopata, Inotawa. PE	192	-12.8092, -69.3182	MG041824	MG041945	MG041886
AMNHA 139315	*D. kamagarini*	Acre, Rio Branco-Porto Velho. BRA	160	-9.9556	-67.8648	AY843652	NA	NA
CORBIDI 8861	*D. kubricki*	Picota, Chambira. PE	679	-7.0375, -76.0900	MG041825	MG041946	MG041887
CORBIDI 6235	*D. kubricki*	Requena, Jenaro Herrera. PE	500	-4.9080, -73.6669	MG041826	MG041947	MG041888
CORBIDI 15778	*D. kubricki*	Requena, Río Tapiche. PE	120	-7.1914, -73.9781	NA	MG041948	NA
QCAZ 43835	*D. brevifrons*	Orellana, Primavera. EC	244	-0.4443, -76.7868	KT721809	NA	NA
QCAZ 28273	*D. brevifrons*	Sucumbíos, Cuyabeno. EC	230	-0.2415, -75.9305	KT721806	NA	NA
QCAZ 17826	*D. brevifrons*	Orellana, Yasuní. EC	245	-0.6782, -76.396	KT721824	NA	NA
ANDES A1025	*D. frosti*	Amazonas, Leticia-Tarapacá km 11. CO	103	-4.1067, -69.9493	JQ088283	NA	NA
CORBIDI 5217	*D. koechlini*	Tambopata, Tambopata. PE	233	-13.1343, -69.6090	MG041827	MG041949	MG041889
CORBIDI 5235	*D. koechlini*	Tambopata, Inotawa. PE	192	-12.6537, -69.1796	MG041828	MG041950	MG041890
QCAZ 10594	*D. marmoratus*	Orellana, Yasuní. EC	250	-0.6744, -76.3971	MG041829	MG041951	MG041891
CFBH 7600	*Xenohyla truncata*	Rio de Janeiro, Restinga de Maricá. BRA	9	-22.9419, -42.8266	AY843775	NA	NA
